# SNP–SNP Interactions of Surfactant Protein Genes in Persistent Respiratory Morbidity Susceptibility in Previously Healthy Children

**DOI:** 10.3389/fgene.2022.815727

**Published:** 2022-03-24

**Authors:** Chintan K. Gandhi, Neal J. Thomas, Ye Meixia, Debbie Spear, Chenqi Fu, Shouhao Zhou, Rongling Wu, Garrett Keim, Nadir Yehya, Joanna Floros

**Affiliations:** ^1^ Center for Host Defense, Inflammation, and Lung Disease (CHILD) Research, Department of Pediatrics, Pennsylvania State University College of Medicine, Hershey, PA, United States; ^2^ Center for Computational Biology, College of Biological Sciences and Technology, Beijing Forestry University, Beijing, China; ^3^ Public Health Science, Pennsylvania State University College of Medicine, Hershey, PA, United States; ^4^ Department of Pediatrics, University of Pennsylvania Perelman School of Medicine, Philadelphia, PA, United States; ^5^ Department of Obstetrics and Gynecology, Pennsylvania State University College of Medicine, Hershey, PA, United States

**Keywords:** persistent respiratory morbidity, long-term outcomes of pediatric acute respiratory failure, SNP–SNP interaction, surfactant protein genetic variant, pediatric acute respiratory failure

## Abstract

We studied associations of persistent respiratory morbidity (PRM) at 6 and 12 months after acute respiratory failure (ARF) in previously healthy children with single-nucleotide polymorphisms (SNPs) of surfactant protein (SP) genes. Of the 250 enrolled subjects, 155 and 127 were followed at 6 and 12 months after an ARF episode, respectively. Logistic regression analysis and SNP–SNP interaction models were used. We found that 1) in the multivariate analysis, an increased risk at 6 and 12 months was associated with rs1124_A and rs4715_A of *SFTPC*, respectively; 2) in a single SNP model, increased and decreased risks of PRM at both timepoints were associated with rs1124 of *SFTPC* and rs721917 of *SFTPD*, respectively; an increased risk at 6 months was associated with rs1130866 of *SFTPB* and rs4715 of *SFTPC*, and increased and decreased risks at 12 months were associated with rs17886395 of *SFTPA2* and rs2243639 of *SFTPD*, respectively; 3) in a two-SNP model, PRM susceptibility at both timepoints was associated with a number of intergenic interactions between SNPs of the studied SP genes. An increased risk at 12 months was associated with one intragenic (rs1965708 and rs113645 of *SFTPA2*) interaction; 4) in a three-SNP model, decreased and increased risks at 6 and 12 months, respectively, were associated with an interaction among rs1130866 of *SFTPB*, rs721917 of *SFTPD*, and rs1059046 of *SFTPA2*. A decreased risk at 6 months was associated with an interaction among the same SNPs of *SFTPB* and *SFTPD* and the rs1136450 of *SFTPA1*. The findings revealed that SNPs of all *SFTP*s appear to play a role in long-term outcomes of ARF survivors and may serve as markers for disease susceptibility.

## Introduction

Acute respiratory failure (ARF) is a common cause of invasive mechanical ventilation need and admission to pediatric intensive care units (PICUs) in children with an incidence of 3% of total PICU admissions (Ibiebele et al., 2018; Khemani et al., 2019). Recent advances in critical care that use early lung protective strategies and improvement in supportive care have led to a gradual decrease in mortality of pediatric ARF (Matthay et al., 2017). This has shifted the focus from mortality to new morbidities in this cohort (Keim et al., 2018). Studies have shown a significant decline in the functional status of pediatric ARF survivors at discharge (Pollack et al., 2009). More specifically, persistent respiratory morbidity (PRM) occurred after 6 and 12 months of an ARF episode even in previously healthy children (Keim et al., 2020). In addition, there is a considerable heterogeneity in the progression of the disease and long-term outcomes of pediatric ARF patients (Keim et al., 2020), indicating a complex interaction between genetic and environmental factors. Nonetheless, studies of long-term sequelae of ARF in children are limited (Yehya and Thomas, 2016). To our knowledge, no studies have specifically examined the role of genetics, an important host variable, as a risk factor for PRM after an episode of ARF in previously healthy children.

Pulmonary surfactant consists of 90% lipids and 10% surfactant proteins (SPs). There are two major types of SPs in the lung; 1) The hydrophobic surfactant proteins (SP-B and -C) are responsible for reducing the surface tension and essential for normal lung function (Serrano and Perez-Gil, 2006), and 2) the hydrophilic SPs (SP-A and -D) are responsible primarily for innate immunity and host defense against infections (Wright, 2005; Kishore et al., 2006; Depicolzuane et al., 2021; Floros et al., 2021). SP-B, SP-C, and SP-D are each encoded by a single gene, *SFTPB*, *SFTPC*, and *SFTPD*, respectively, whereas SP-A is encoded by two similar genes, *SFTPA1* and *SFTPA2*, that are differentially regulated (Floros and Tsotakos, 2021) and identified with functional, structural, and other differences (Thorenoor et al., 2019; Gandhi et al., 2020b; Thorenoor et al., 2020; Xu et al., 2020; Floros et al., 2021). Several single-nucleotide polymorphisms (SNPs) have been described for each of these genes (DiAngelo et al., 1999; Wert et al., 2009; Silveyra and Floros, 2012). These SNPs are common in the general population and shown to associate with various acute and chronic pulmonary diseases, such as neonatal respiratory distress syndrome (RDS) (Kala et al., 1998; Nogee et al., 2000; Rämet et al., 2000; Floros et al., 2001), cystic fibrosis (Lin et al., 2018), acute respiratory distress syndrome (Lin et al., 2000b), chronic obstructive pulmonary disease (Seifart et al., 2002), interstitial pulmonary fibrosis (Selman et al., 2003), severity of respiratory syncytial virus (RSV) (Thomas et al., 2009), tuberculosis (TB) (Floros et al., 2000), and hypersensitivity pneumonitis (HP) (Gandhi et al., 2021). Importantly, we previously demonstrated that these SNPs are associated with pediatric ARF and its short-term outcome, pulmonary dysfunction. at discharge in the same cohort (Gandhi et al., 2020a).

Taken together, we postulated that the SPs contribute to the progression of pediatric ARF and its long-term outcome, PRM, at 6 and 12 months after the index admission for ARF. To eliminate potential confounding contribution of other chronic illnesses to long-term sequelae of pediatric ARF, we enrolled only previously healthy children for the current study. We hypothesized that multiple genetic variants of the SP genes are associated with long-term outcomes after an ARF episode through single genetic variations within a gene, and/or through intragenic (within the same gene) or intergenic (with different genes) interactions. To our knowledge, this is the first study examining the association of genetic variants in PRM after an admission for ARF in previously healthy children. Our results indicate the association of complex SNP–SNP interactions of the surfactant protein genes with PRM at 6 and 12 months, and may contribute to the pulmonary sequelae in pediatric ARF survivors.

## Subjects and Methods

### Study Population

We prospectively enrolled 250 previously healthy children from 0 to 24 months of age that required invasive ventilation for an index case of ARF secondary to respiratory illness at 10 participating pediatric intensive care units (PICUs) over 5 consecutive years. This multicenter cohort has been described in detail elsewhere (Gandhi et al., 2020a; Keim et al., 2020). Briefly, previously healthy children, who met at least one of the three criteria, 1) chest radiograph with either focal or diffuse infiltrative pulmonary process, 2) radiographic evidence of air trapping, or 3) clinical exam findings of lower respiratory tract illness, were determined to have primary respiratory cause of ARF. We prospectively collected all demographic and clinical data for children with ARF.

These subjects were followed up at 6 (*n* = 155) and 12 months (*n* = 127) after the index ARF admission via telephonic interview of a designated parent about the subject’s health status. Questions included the 11-item PedsQL™ asthma module health-related quality of life symptom scale (Chan et al., 2005; Greenley et al., 2008; Seid et al., 2010). The parents’ responses were recorded on a scale of 0–4, where 0 = never and 4 = almost always. Parents were also asked about prescribed medications, frequency of use, and whether the child had been diagnosed with asthma, and/or had visits to the physician’s office or emergency department or had been readmitted to the hospital, PICU for “breathing problems,” and finally, if the child required mechanical ventilation post index admission.

Cases: children, at 6 and 12 months of discharge, who developed PRM as defined *a priori,* i.e., if the subject met one of the following criteria: 1) diagnosis of asthma, 2) use of bronchodilator in the last month, 3) use of inhaled corticosteroid, 4) representation to care for a “breathing”-related complaint, or 5) asthma module health-related quality of life symptom scale score ≥5. The cohort of the current study differs from the original ARF cohort in terms of chronicity and long-term respiratory symptoms. In other words, the initial incident is defined as ARF; however, ∼45% of the ARF children continue to have breathing symptoms and get diagnosed with PRM. Thus, all children with PRM had an episode of ARF, but not all patients with ARF developed PRM. Controls: children who did not meet predefined criteria of PRM at 6 and 12 months following an index admission to PICU for ARF.

We collected blood samples of the study participants after obtaining informed consent from a parent or legal guardian. This study was approved by the institutional review board of participating sites.

### DNA Isolation and Genotype Analysis

Genomic DNAs were extracted from blood samples using QIAamp Blood kit (Qiagen, Valencia, CA, USA) as described previously (DiAngelo et al., 1999). We used the polymerase chain reaction-restriction fragment length polymorphism (PCR-RFLP) method to analyze the *SFTPA1*, *SFTPA2*, *SFTPD* (DiAngelo et al., 1999; Lin et al., 2000b), *SFTPB* (Lin et al., 2000a; Lin et al., 2000b), and *SFTPC* (Selman et al., 2003) gene polymorphisms as described earlier (DiAngelo et al., 1999). The PCR primer sequences and restriction enzymes used for the current study are described elsewhere (DiAngelo et al., 1999; Gandhi et al., 2020a; Gandhi et al., 2021). A total of 14 target SNPs of surfactant protein genes *SFTPA1*, *SFTPA2*, *SFTPB*, *SFTPC*, and *SFTPD* were selected based on their associations with various acute and chronic pulmonary diseases (Lin et al., 2000b; Floros et al., 2000; Floros et al., 2001; Selman et al., 2003; Thomas et al., 2009; Silveyra and Floros, 2012; Lin et al., 2018; Gandhi et al., 2020a; Gandhi et al., 2021). These include: five SNPs from *SFTPA1*: rs1059047, rs1136450, rs1136451, rs1059057, and rs4253527; four SNPs from *SFTPA2*: rs1059046, rs17886395, rs1965707, and 1965708; one SNP from *SFTPB*: rs1130866; two SNPs from *SFTPC*: rs4715 and rs1124; and two SNPs from *SFTPD*: rs721917 and rs2243639. The details of the studied SNPs are given in Supplementary Table S1. The SP-A1 and SP-A2 genotypes were assigned as described (DiAngelo et al., 1999). To reduce bias in the genotype, all samples were processed together in a blinded fashion with those assigning genotypes unaware of the clinical status.

### Statistical Analysis

The frequency of the alleles in the two groups were compared using the Chi-square test, or the Fisher’s exact test when the expected frequency of the allele was too small (<5). Assuming no allele dose–effect, univariate logistic regression was applied to each allele or SP-A genotype to test whether the existence of a given minor allele and/or genotype distinguishes PRM from no PRM. Alleles that were significantly associated with PRM in univariate analysis (*p*-value < 0.1) were included in the multivariate logistic regression analysis (Floros et al., 2000; Selman et al., 2003). The univariate analysis was done for screening and selection of variables for the multivariate analysis; therefore, the relaxed *p*-value of less than 0.1 was used. In the multivariate analysis of PRM at 12 months, a positive bacterial culture on admission and PRM at 6 months were obliged to be included in the model due to their significant associations in the univariate analysis. Variable selection was performed using a backward elimination method with a prespecified significance level of 0.05.

Wang et al. (2010) developed a computational model for detecting additive, dominant, and epistatic effects by integrating quantitative genetic theory into a case-control design context. This model can particularly characterize high-order epistatic interactions even with the modest sample size; hence, we used this model (Wang et al., 2010) to study associations of SP gene polymorphisms with PRM at 6 and 12 months (Wang et al., 2010; Gandhi et al., 2020a; Gandhi et al., 2021). Of note, in the present study, the reference (major) and alternate (minor) alleles were assigned based on the “NCBI dbSNP database of genetic variation” using the global population (Sherry et al., 2001), and the significant findings were noted in terms of the reference to the minor allele in its homozygous or heterozygous form. The model of Wang et al. dissects the genetic effects, including the additive (a) and dominant (d) of the minor allele at a single SNP, pairwise interaction effects at two SNPs, and three-way interactions in a three-SNP model.

An example with a detailed explanation is provided below in order to understand the additive and dominant effects of each SNP in a given interaction type. Please consider the example of an SNP with three genotypes AA, Aa, and aa. To estimate its additive effect, the homozygotes (AA and aa) were compared against its heterozygote (Aa), whereas to estimate its dominant effect, the heterozygote (Aa) was compared against the average size of the two homozygotes (AA and aa). Thus, the interaction type “a1d2” in a two-SNP model [with the first SNP with three possible genotypes (AA, Aa, and aa) and the second SNP with three possible genotypes (BB, Ba, and bb)] can be interpreted as follows: two-locus genotypes with a homozygote at the first locus and heterozygote at the second locus, i.e., AABb, aaBb, perform differently than the remaining genotypes (AABB, AAbb, AaBB, AaBb, Aabb, aaBB, and aabb). According to this model, we sorted the case-control genotype observations into a 2 × 2 contingency table to examine the association of each of the genetic effects of individual SNPs with PRM at 6 and 12 months.

The logistic regression model was implemented to estimate the genetic effect of that particular SNP after adjusting for covariates (age, sex, race, and weight). These variables were selected based on the biological possibilities and the significant difference between groups. We used the race as a covariate to adjust for differences in allele frequencies between races. The OR with 95% confidence interval (95% CI) was estimated using the Cochran’s and Mantel–Haenszel tests to assess the magnitude of the dominant/additive effect (Day and Byar, 1979). The false discovery rate (FDR) was controlled at 5% using the Benjamini–Hochberg method to account for multiple testing (Hope, 1968; Hochberg, 1995). We reported all possible SNP–SNP interactions associated with cases with *p*-value < 0.05 for single SNPs and two- and three-SNP interaction models.

## Results

### Clinical Characteristics of the Study Group

**FIGURE 1:**
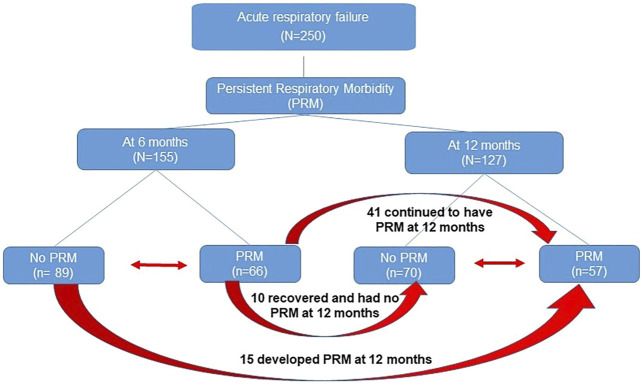
Flow diagram of patients with persistent respiratory morbidity (PRM). Red arrow depicts transfer of patients from one group to another.

Of the 250 patients enrolled in the study, follow-up questionnaires were completed for 155 patients (∼61%) and 127 patients (∼50%) at 6 and 12 months, respectively. Persistent respiratory morbidity was diagnosed in 66 patients (42.5%) at 6 months and in 57 patients (44.8%) at 12 months. Figure 1 shows the flow diagram of patients with PRM. We did not observe statistically significant difference in age, sex, race, and ethnicity between groups at both timepoints as shown in Table 1. As shown previously in our clinical paper (Keim et al., 2020), PRM at 6 months was predictive of developing PRM at 12 months, whereas a positive respiratory bacterial culture during the index admission was predictive of developing PRM at both timepoints.

**TABLE 1:** Demographics and clinical characteristics of the study group at 6 and 12 months.

Variable	At 6 months			At 12 months		
	No PRM (*n* = 89)	PRM (*n* = 66)	*p*-Value	No PRM (*n* = 70)	PRM (*n* = 57)	*p*-Value
Demographics						
Age (months)	3 ± 4.4	4.2 ± 4.8	0.123	2.8 ± 3.9	4.4 ± 5.6	0.07
Female (%)	30 (34)	24 (36)	0.734	23 (33)	21 (37)	0.6
Non-White race (%)	29 (33)	17 (26)	0.177	22 (31)	19 (33)	0.56
Hispanic (%)	14 (16)	14 (21)	0.384	12 (17)	9 (16)	0.84
Admission diagnosis (%)			0.387			0.492
RSV bronchiolitis	50 (56)	36 (54)		37 (53)	32 (56)	
Other bronchiolitis	17 (19)	10 (15)		14 (20)	11 (19)	
Other pneumonia	8 (9)	11 (17)		5 (7)	7 (12)	
Other respiratory failure	11 (12)	9 (14)		12 (17)	6 (11)	
Nonpulmonary	3 (4)	0		2 (3)	1 (2)	
Positive bacterial culture (%)	39 (44)	41 (62)	0.04	26 (37)	36 (63)	0.001
PDAD (%)	19 (21)	31 (47)	0.001	19 (27)	21 (37)	0.245
PRM at 6 months (%)	-	-	-	10 (14)	41 (72)	1.6924E−13

Note. PRM, persistent respiratory morbidity; PDAD, pulmonary dysfunction at discharge; RSV, respiratory syncytial virus.

### Association of Surfactant Protein Single-Nucleotide Polymorphisms With Persistent Respiratory Morbidity

#### Univariate and Multivariate Analyses

At 6 and 12 months, no significant differences were observed in the frequency of the studied SNPs between the two groups (PRM vs. no PRM). The frequency distribution of the majority of SNPs did not deviate from the Hardy–Weinberg equilibrium (Supplementary Tables S2 and S3). An increased risk of PRM at 6 months was significantly associated with rs1124 of the *SFTPC* in the univariate and multivariate logistic regression analysis, OR = 11.7 (1.9–217.9), *p* = 0.03 (Supplementary Table S4). At 12 months, significant differences (*p* < 0.1) were observed for the SFTPA2 marker allele (rs17886395_G), the SF*TPD* marker allele (rs721917_G), the *SFTPC* marker alleles (rs4715_A, rs1124_A), and SFTPA1 (6A3) in the univariate analysis. Of these, based on an OR <1, a decreased risk for PRM was associated with rs721917_G of the *SFTPD* and SFTPA1 (6A3), whereas an increased risk for PRM was associated with other marker alleles (OR >1) (Table 2). When these marker alleles were considered in the multivariate analysis, an increased risk for PRM was significantly associated with only one allele, the *SFTPC* (rs4715_A), OR = 3 (1.14–9.5), *p* = 0.04 (Table 2).

**TABLE 2:** Persistent respiratory morbidity (PRM) vs. no PRM at 12 months (univariate analysis).

Gene	SNP	Chr	Position	Allele	PRM	No PRM	OR (95%CI)	*p*-Value	OR (95%CI)*	*p*-Value*
					*n* (%)	*n* (%)				
*SFTPA2*	rs17886395	10	AA91	G	30 (26)	23 (16)	2 (0.91–4.5)	0.09	2.02(0.70–6.05)	0.2
*SFTPD*	rs721917	10	AA11	G	57 (50)	60 (43)	0.3 (0.11–0.93)	0.04	0.4 (0.10–1.71)	0.2
*SFTPC*	rs4715^**#**^	8	AA138	A	21 (19)	38 (27)	2 (1–4.2)	0.06	3 (1.14–9.5)	0.04
*SFTPC*	rs1124	8	AA186	A	28 (25)	52 (37)	1.9 (1.04–3.6)	0.04	2.4 (1.03–6.2)	0.05
*SFTPA1*	6A^3^	10			34 (25)	23 (21)	0.66 (0.32–1.37)	0.27	0.36 (0.11–1.05)	0.07

Note. Chr, chromosome; AA, amino acid; n (%). number of the given allele, in parenthesis the percentage of the given allele out of the possible alleles in the particular cohort is shown. *adjusted for PRM at 6 months and positive bacterial culture. ^**#**^remained significant in the multivariate analysis. OR, odds ratio; CI, confidence interval.

#### Single-Single-Nucleotide Polymorphism Model

At 6 and 12 months, an increased risk of PRM was associated with rs1124, OR = 5.8 (1.8–19.3) of the *SFTPC* that exhibited an additive effect, whereas a decreased risk of PRM was associated with rs721917 of the *SFTPD* that exhibited a dominant effect, *p* < 0.05. Only at 6 months, an increased risk of PRM was associated with rs1130866 of the *SFTPB*, OR = 3.2 (1.2–8.6), and the rs4715 of the *SFTPC*, OR = 6.2 (1.4–27.4), and each exhibited an additive effect. Only at 12 months, increased and decreased risks of PRM were associated with rs17886395 of the *SFTPA2* and rs2243639 of the *SFTPD*, respectively, and exhibited a dominant effect (Table 3).

**TABLE 3:** Association of surfactant protein (SP) gene single-nucleotide polymorphisms (SNPs) with persistent respiratory morbidity (PRM) at 6 and 12 months in a single-SNP model after adjusting for covariates (age, sex, race, and weight).

SNP	Gene	Allele	Interaction type	PRM at 6 months			PRM at 12 months		
				*p*-Value	FDR	OR (95%CI)	*p*-Value	FDR	OR (95%CI)
rs1124	*SFTPC*	A	Additive	3.20E−03	0.02	5.8 (1.8–19.3)	0.0025	0.02	6.1 (1.9–19.8)
rs721917	*SFTPD*	G	Dominant	9.00E−04	0.01	0.5 (0.3–0.8)	3.51E−05	0.001	0.3 (0.2–0.5)
rs1130866	*SFTPB*	C	Additive	4.00E−04	0.01	3.2 (1.2–8.6)			
rs4715	*SFTPC*	A	Additive	4.30E−03	0.02	6.2 (1.4–27.4)			
rs17886395	*SFTPA2*	G	Dominant				0.001	0.02	2.0 (1.1–3.8)
rs2243639	*SFTPD*	C	Dominant				0.001	0.02	0.4 (0.2–0.8)

Note. OR, odds ratio; CI, confidence interval; FDR, false discovery rate.

For SNP–SNP interaction tables, the column “interaction type” represents interactions that could be intragenic, i.e., between SNPs of an individual gene, or intergenic, i.e., between SNPs of different genes. The letter “a” is for additive and “d” is for dominant effect of that particular SNP. The number following “a” or “d” indicates the position of the corresponding SNP, for example, an interaction of the a1d2 type indicates the additive and dominant effects of SNP 1 and SNP 2, respectively, in the two-SNP model. For the three-SNP model, the a1a2d3 interaction type indicates the additive effects of SNPs 1 and 2, and the dominant effect of SNP 3.

#### Two-Single-Nucleotide Polymorphism Model

At 6 months, decreased risk of PRM was associated with 12 interactions of different combinations between SNPs of the studied genes in a two-SNP model, OR = 0.1–0.5 (Table 4). All interactions were intergenic (between SNPs of different genes). The majority of significant interactions involved the rs1130866 of the *SFTPB* (*n* = 7) and interacted with SNPs of hydrophilic SPs (*n* = 6). We observed four and two interactions between SNPs of the hydrophilic and hydrophobic SPs alone, respectively.

**TABLE 4:** Associations of SP gene SNP interactions with persistent respiratory morbidity (PRM) at 6 months in a two-SNP model after adjusting for covariates (age, sex, race, and weight).

SNP#1	Gene	SNP#2	Gene	Interaction type	*p*-Value	FDR	OR (95% CI)
rs1136451	*SFTPA1*	rs721917	*SFTPD*	a1	3.27E−04	0.0020	0.1 (0.1–0.4)
				d2	3.05E−05	0.0007	0.5 (0.2–0.9)
rs1130866	*SFTPB*	rs2243639	*SFTPD*	a1	1.55E−03	0.0050	0.2 (0.1–0.9)
				d1d2	6.68E−06	0.0006	0.5 (0.3–0.8)
rs1965707	*SFTPA2*	rs1130866	*SFTPB*	a2	2.12E−04	0.0017	0.3 (0.1–0.9)
rs1136451	*SFTPA1*	rs1130866	*SFTPB*	a2	9.22E−05	0.0011	0.2 (0.1–0.7)
rs1965707	*SFTPA2*	rs1136450	*SFTPA1*	a1d2	6.26E−05	0.0010	0.4 (0.2–0.9)
rs1136450	*SFTPA1*	rs2243639	*SFTPD*	a1d2	1.65E−03	0.0051	0.3 (0.1–0.8)
				a1a2	3.12E−02	0.0430	0.2 (0.1–0.8)
rs1130866	*SFTPB*	rs4715	*SFTPC*	a1d2	3.14E−05	0.0007	0.3 (0.1–0.6)
rs1130866	*SFTPB*	rs1124	*SFTPC*	a1d2	5.85E−06	0.0006	0.3 (0.1–0.8)
rs1130866	*SFTPB*	rs721917	*SFTPD*	a1d2	3.46E−04	0.0020	0.5 (0.2–0.9)
rs1965708	*SFTPA2*	rs721917	*SFTPD*	d1d2	5.88E−04	0.0027	0.6 (0.4–0.9)
rs1059046	*SFTPA2*	rs1130866	*SFTPB*	a2	5.74E−03	0.0131	0.2 (0.1–0.8)
rs1059046	*SFTPA2*	rs721917	*SFTPD*	d2	6.92E−03	0.0150	0.4 (0.2–0.8)

Note. Interaction type: a and d denote additive and dominant effects of the particular SNP. Numbers 1 and 2 denote effect of the particular SNP at that position. For example, the rs1965707 × rs1136450 interaction is a1d2 type indicating an additive effect of the rs1965707 and a dominant effect of the rs1136450. This interaction is associated with a decreased risk of PRM at 6 months. In some interactions, only one SNP exhibited a main effect, whereas, the other SNP remained silent but their interaction was significant. For example, the rs1136451 × rs721917 interaction shows two significant effect types a1 and d2 with the rs1136451 exhibiting a main additive effect and the rs72191 exhibiting a dominant effect.

At 12 months, PRM was associated with a total of 29 interactions among SNPs of SP genes in a two-SNP model (Table 5). All but one interactions were intergenic. The one intragenic interaction (SNPs of the same gene) was between SNPs of the *SFTPA2* (rs1059046 × rs1965707, a1d2, OR = 2.9 (1.1–7.8), *p* < 0.05). Significant intergenic interactions (*n* = 28) that included the other studied genes were as follows: 15, 10, 8, and 5 interactions for each *SFTPA1*, *SFTPA2*, *SFTPB*, and *SFTPC*, respectively. The *SFTPD* SNPs had the highest number of interactions with SNPs of other SPs (*n* = 18), particularly the rs721917 of the *SFTPD* (*n* = 13).

**TABLE 5:** Associations of SP gene SNPs with persistent respiratory morbidity (PRM) at 12 months in a two-SNP model after adjusting for covariates (age, sex, race, and weight).

SNP#1	Gene	SNP#2	Gene	Interaction type	*p*-Value	FDR	OR (95% CI)
rs1059046	*SFTPA2*	rs1124	*SFTPC*	d1a2	4.92E−04	0.001051	0.5 (0.3–0.9)
rs1130866	*SFTPB*	rs1124	*SFTPC*	d1a2	1.68E−05	0.000102	0.4 (0.2–0.7)
rs1136450	*SFTPA1*	rs1124	*SFTPC*	a2	8.50E−05	0.000288	0.1 (0.1–0.6)
				d1a2	3.33E−04	0.000792	0.6 (0.3–0.9)
rs1965707	*SFTPA2*	rs1124	*SFTPC*	a2	8.62E−04	0.001716	0.2 (0.1–0.6)
rs1059046	*SFTPA2*	rs1130866	*SFTPB*	a2	5.38E−03	0.007326	0.3 (0.1–0.9)
rs1136451	*SFTPA1*	rs1130866	*SFTPB*	d1d2	5.35E−06	5.83E−05	0.6 (0.4–0.9)
rs1965707	*SFTPA2*	rs1130866	*SFTPB*	a2	1.61E−05	0.000101	0.3 (0.1–0.7)
				a1d2*	4.38E−05	0.000165	1.8 (1.1–3.1)
rs1965707	*SFTPA2*	rs1136450	*SFTPA1*	a1d2	9.22E−07	1.91E−05	0.4 (0.2–0.7)
				a1*	5.17E−04	0.001092	6.6 (1.9–22.6)
				d1a2	1.22E−02	0.014944	0.5 (0.3–0.9)
rs1965708	*SFTPA2*	rs1136450	*SFTPA1*	a1d2*	1.74E−03	0.003022	1.8 (1.1–3.1)
				d1a2	5.20E−03	0.007177	0.5 (0.3–0.9)
**rs1059046**	***SFTPA2***	**rs1965707**	***SFTPA2***	a1d2*	**2.31E−03**	**0.003708**	**1.9 (1.1–3.5)**
rs1124	*SFTPC*	rs2243639	*SFTPD*	d2	1.27E−03	0.002345	0.5 (0.3–0.8)
rs1130866	*SFTPB*	rs2243639	*SFTPD*	d1d2	2.38E−08	1.64E−06	0.5 (0.3–0.7)
				a1	4.38E−04	0.000965	0.2 (0.1–0.6)
				a2*	2.99E−03	0.004446	4.5 (1.2–16.4)
rs1136450	*SFTPA1*	rs2243639	*SFTPD*	a1d2	1.53E−04	0.000421	0.3 (0.2–0.6)
rs4715	*SFTPC*	rs2243639	*SFTPD*	d2	1.05E−04	0.000328	0.6 (0.4–0.9)
rs1130866	*SFTPB*	rs4715	*SFTPC*	d1a2	6.21E−04	0.001261	0.6 (0.3–0.9)
rs1059046	*SFTPA2*	rs721917	*SFTPD*	d2	1.98E−05	0.000103	0.3 (0.2–0.5)
				a1a2*	3.13E−03	0.004568	4.1 (1.3–13.6)
				a1d2*	9.75E−03	0.012155	1.9 (1.1–3.2)
rs1124	*SFTPC*	rs721917	*SFTPD*	a1	3.13E−05	0.000145	0.1 (0.1–0.8)
				d2	3.62E−04	0.000848	0.5 (0.3–0.8)
				d1d2	4.25E−05	0.000165	0.7 (0.5–0.9)
rs1130866	*SFTPB*	rs721917	*SFTPD*	a1d2	3.10E−06	4.35E−05	0.4 (0.2–0.7)
				d2	3.45E−05	0.000149	0.4 (0.2–0.8)
				d1a2	5.93E−04	0.001227	0.5 (0.3–0.9)
				d1d2	4.34E−05	0.000165	0.7 (0.5–0.9)
rs1136450	*SFTPA1*	rs721917	*SFTPD*	d2	4.24E−04	0.000963	0.4 (0.2–0.8)
				a1a2	1.49E−03	0.002732	0.1 (0.1–0.4)
rs1136451	*SFTPA1*	rs721917	*SFTPD*	a1	3.15E−06	4.35E−05	0.1 (0.1–0.3)
				d2	5.01E−09	1.01E−06	0.3 (0.2–0.6)
				a1d2	3.99E−07	1.03E−05	0.5 (0.3–0.9)
				d1d2*	6.90E−05	0.000251	1.6 (1.2–2.3)
rs1965707	*SFTPA2*	rs721917	*SFTPD*	d2	1.10E−05	7.60E−05	0.4 (0.3–0.8)
				a1d2	1.84E−05	0.000103	0.5 (0.3–0.8)
rs1965708	*SFTPA2*	rs721917	*SFTPD*	d1d2	3.50E−06	4.42E−05	0.6 (0.5–0.9)
				d2	3.69E−03	0.005302	0.5 (0.3–0.9)
				a1d2*	6.47E−03	0.008473	1.8 (1.1–3.1)
rs4715	*SFTPC*	rs721917	*SFTPD*	d2	7.89E−05	0.000277	0.5 (0.3–0.8)
				d1d2	3.14E−05	0.000145	0.7 (0.5–0.9)

Note. Interaction type: a and d denote additive and dominant effects of the particular SNP. Numbers 1 and 2 denote the effect of the particular SNP at that position. An intragenic interaction is shown in bold. Interaction type that is associated with increased risk of PRM is marked with “*”. In some interactions, only one SNP exhibited a main effect, whereas the other SNP remained silent, but their interaction was significant. For example, the rs1136450 × rs1124 interaction is an a2 type, indicating that in this interaction, the main additive effect of rs1124 is significant.

Out of the 29 interactions, 11 were among SNPs of both hydrophilic and hydrophobic SPs, and 17 and 1 were between SNPs of the hydrophilic and hydrophobic SPs alone, respectively. A decreased risk of PRM was associated with the majority of the interactions, whereas an increased risk of PRM was associated with only 10 interactions. Of note, the susceptibility to PRM changed based on the effect of a particular SNP in a given interaction. For example, if the increased risk of PRM was associated with the interaction (rs1059046 × rs1124), and the interaction type was a1a2, OR = 5.3 (1.1–25.1), it would indicate that the additive effects of both SNPs were associated with increased risk. However, the decreased risk of PRM was associated with the same interaction, if the interaction type was d1a2, OR = 0.4 (0.2–0.8), this indicates that the dominant effect of rs1059046 and the additive effect of rs1124 are associated with decreased risk. In addition, 9 out of the 12 significant interactions associated with a decreased risk of PRM at 6 months remained significant at 12 months as well.

#### Three-Single-Nucleotide Polymorphism Model

At 6 and 12 months, the rs1130866 of the *SFTPB*, and the rs721917 of the *SFTPD* interacted with the rs1059046 of the *SFTPA2* in a three-SNP model (Table 6). A decreased risk of PRM at 6 months was associated with these intergenic interactions. However, an increased risk of PRM at 12 months was associated with the same interactions. In addition, a decreased risk of PRM only at 6 months was associated with interactions among the same SNPs of the *SFTPB* and *SFTPD* (noted above) and the rs1136450 of the *SFTPA1*. Furthermore, as shown in Table 6, the effect size of the seven intergenic interactions. as denoted by the OR, was variable, based on the effect (additive or dominant) of the particular SNP at the particular position, OR = 0.07–0.25 (Table 6).

**TABLE 6:** Association of SP gene SNP interactions with persistent respiratory morbidity (PRM) at 6 and 12 months in a three-SNP model after adjusting for covariates (age, sex, race, and weight).

SNP#1	SNP#2	SNP#3	PRM at 6 months				PRM at 12 months			
			Interaction type	*p*-Value	FDR	OR (95%CI)	Interaction type	*p*-Value	FDR	OR (95%CI)
rs1059046	rs1130866	rs721917	a1a2d3	0.00E + 00	0.0001	0.07 (0.02–0.19)	a1d3	6.21E−03	0.01	5.5 (1.5–20.5)
			a2d3	2.30E−04	0.002	0.21 (0.07–0.62)	a2d3	2.33E−03	0.005	4.5 (1.4–14.6)
			d2a3	1.08E−03	0.005	0.25 (0.09–0.72)	d2a3	7.70E−03	0.01	3.8 (1.1–13.7)
			d2	5.20E−04	0.003	0.14 (0.04–0.44)				
rs1136450	rs1130866	rs721917	d2	9.00E−05	0.001	0.10 (0.03–0.35)
			d2a3	2.20E−04	0.002	0.24 (0.08–0.72)

Note. Interaction type: a and d denote additive and dominant effects of the particular SNP. Numbers 1, 2, and 3 denote the effect of the particular SNP at that position. For example, the rs1136450 × rs1130866 × rs721917 interaction exhibits two effect types, d2 and d2a3. This indicates that the main dominant effect of rs1130866 in d2 type and the dominant and additive effects of rs1130866 and rs721917, respectively, in the d2a3 type, are each significant. In the d2a3 interaction, the rs1136450 remained silent.

## Discussion

Surfactant dysfunction and dysregulated inflammation, individually or in conjunction with each other, are central to the pathophysiologic mechanisms of various pulmonary diseases, including ARF in children (Amigoni et al., 2017). Because SPs play a role in surfactant dysfunction and/or regulation of inflammatory processes/innate immunity, we hypothesized that natural genetic variants of SPs are associated with PRM at 6 and 12 months after an ARF episode. The results indicated that 1) PRM at both timepoints is associated with SNPs of all five SP genes. 2) Increased risk of PRM is associated with rs1124 of the *SFTPC* at 6 months in the univariate and multivariate analyses and in the single-SNP model, whereas increased risk of PRM at 12 months is associated with rs4715 of the *SFTPC* in the univariate and multivariate analyses. 3) At both timepoints, increased and decreased risks of PRM is associated with rs1124 of the *SFTPC* and the rs721917 of the *SFTPD*, respectively, in the single-SNP model. 4) Increased and decreased risks of PRM at 12 months is associated with rs17886395 of the SFTPA2 and rs2243639 of the *SFTPD*, respectively, in the single-SNP model. 5) PRM at 12 months is associated with one significant intragenic interaction between SNPs of the *SFTPA2* (rs1059046 × rs1965707). 6) No association between increased and decreased risks of PRM at 6 and 12 months, respectively, was observed with any of the SNP−SNP interactions in the two- and three-SNP model (6 months) or the three-SNP model (12 months). 7) In the three-SNP model, one intergenic interaction (rs1059046 × rs1130866 × rs721917) is associated with decreased and increased risk of PRM at 6 and 12 months, respectively. 8) The intergenic (rs1136450 × rs1130866 × rs721917) interaction is associated with a decreased risk of PRM at 6 months with a variable effect size.

We used two different statistical methods to study associations of SNPs of the SP genes with PRM at 6 and 12 months. The first one is the multivariate logistic regression analysis adjusting for selected covariates using backward elimination method (*p* < 0.1). The other method is the Wang’s SNP–SNP interaction model, an integrated approach, which uses principles of quantitative genetics to decompose the genetic effect of a particular SNP into its underlying components (Wang et al., 2010). In this analysis, the covariates (age, sex, race, and weight) were selected based on the biological possibilities and the differences between the two groups (cases vs. controls). The marker alleles shown to associate with risk of PRM at 6 and 12 months are almost identical (based on the OR) to those observed in the univariate and multivariate analyses. These observations indicate that these associations are true rather than spurious and may validate the newer two- and three-SNP–SNP interaction models.

Association of SP SNPs in the single-SNP model: In the single-SNP model, decreased risk of PRM at both timepoints was associated with rs721917 of the *SFTPD* (Table 3). The rs721917 results in an alteration of the codon corresponding to amino acid 11 in the mature protein, where a methionine is replaced by a threonine. The Thr11 (C allele) variant has been associated with low serum levels of SP-D (Heidinger et al., 2005) and is shown to inhibit SP-D oligomerization (Heidinger et al., 2005). Previously, this SNP (C allele) is shown to associate with an increased risk of severe RSV (Lahti et al., 2002) and TB (Floros et al., 2000). In this study, we found the rs721917_G allele to associate with a decreased risk of PRM, which is consistent with the previous findings, where the C allele was associated with an increased risk. However, the difference in allele significance among studies may partly be due to differences in study populations and disease processes.

Increased risk of PRM at both timepoints was associated with rs1124 of the *SFTPC* (Table 3), whereas increased risk of PRM at 6 months was associated with the rs4715 (A allele) of the *SFTPC* only. Previously, we showed in the same dataset (Gandhi et al., 2020a) that the rs4715 (A allele) was associated with an increased risk of ARF (compared with nonARF newborns) but not with the short-term outcome, pulmonary dysfunction at discharge. Other studies have shown that haplotypes of these SNPs, but not of individual SNPs, are associated with severity of RSV infection but are protective against the long-term outcome, asthma (Puthothu et al., 2006). Although in the current study, haplotype analysis was not performed; this is a goal in future studies. Conversely, although 55% of the children in our study had RSV bronchiolitis, an increased risk of PRM was associated with each of the *SFTPC* SNPs. These contrasting findings could be due to difference in patient population, environmental conditions, case-control definitions, and/or statistical approaches used for the studies. To date, no studies have been done to examine the functional impact of these polymorphisms. Therefore, we can only speculate at this time. A preclinical study in mice has shown that SP-C encoded by the *SFTPC* gene is important for stabilization and recruitment of phospholipids in surfactant (Glasser et al., 2001). It is plausible that these polymorphisms may decrease surfactant stability and, in turn, increase susceptibility to PRM.

Increased risk of PRM at 6 months was associated with the rs1130866 (C allele) of *SFTPB.* The same SNP is shown to associate with an increased risk of various other pulmonary diseases, such as chronic obstructive pulmonary disease (Seifart et al., 2002), acute respiratory distress syndrome (Lin et al., 2000b), interstitial pulmonary fibrosis (Selman et al., 2003), and ARF in adults (Quasney et al., 2004), but with a decreased risk of HP (Gandhi et al., 2021) and neonatal RDS (Floros et al., 2001). This SNP is shown to increase apoptosis, lung injury, and mortality in humanized transgenic mice (Xu et al., 2016). Moreover, this SNP (rs1130866) is part of an N-linked glycosylation site [Asn(129)-Gln-Thr131] enabling posttranscriptional N-linked glycosylation of proSP-B (Wang et al., 2003). An *in vitro* study showed an allele-specific (Ile131Thr) delay in the secretion of SP-B as well as a lower rate of secretion under experimental conditions (Taponen et al., 2013). Furthermore, a transgenic mouse model of pneumonia and sepsis carrying the C allele of this SNP showed a decreased number of lamellar bodies, SP-B concentration, and increased surface tension compared with wild-type mice after infection (Yang et al., 2019). These biologic mechanisms may shed light on the association of the rs1130866 with increased risk of PRM in our patient population where the most common etiology of ARF was pneumonia. In summary, given the importance of SP-B and SP-C in normal lung function, we postulate that SNPs of the hydrophobic proteins play a central role in ARF and its disease progression even after 1 year of the initial insult in previously healthy children. These SNPs, although not part of the mature protein, may modulate various aspects of the encoded precursor proteins, function, or other, as discussed above for SP-B, although the mechanistic details are currently unknown.

Increased risk of PRM only at 12 months was associated with rs17886395 (G allele) of the *SFTPA2* gene in the single-SNP model. In contrast, the same SNP (G allele) was associated with a decreased risk of community-acquired pneumonia in Spanish adults (García-Laorden et al., 2011). Of note, the same SNP (G allele) was associated with an increased risk of TB and allergic bronchopulmonary aspergillosis in Indian study groups (Madan et al., 2002; Saxena et al., 2003). This SNP changes the amino acid from proline (C allele) to alanine (G allele). Proline is an important component of the repetitive subunit Gly-X-Pro in the collagen region of SP-A and is known to provide stability to triple helical collagenous structures (Improta et al., 2001). We speculate that this SNP (G allele) leads to unstable and/or partially functional SP-A, and this, in turn, may increase susceptibility to respiratory infections.

Interestingly, the majority of the significant SNPs in the single-SNP model are associated with increased risk of PRM; however, when found in interactions with other SNPs, they are associated with a decreased risk of PRM at both timepoints. Recent studies have shown that a genetic variant in the presence of another variant can alter the susceptibility of an individual to certain diseases (Cordell, 2009). The additive and/or epistatic interactions among surfactant protein genetic variants may alter concentrations and/or functional capabilities of certain SPs, and/or host defense at the cellular, molecular, or tissue level (Cordell, 2009). In addition, we have previously shown association of SP SNP interactions (but not with a single SNP) with ARF and its short-term outcome (Gandhi et al., 2020a). Collectively, our results support that epistasis plays an important role in the development and progression of complex diseases, such as PRM (Marchini et al., 2005), and studying SNP–SNP interactions is crucial to our understanding of the regulation of physiological function and their impact in health and disease state.

Association of SP SNPs in the two- and three-SNP model: Decreased risk of PRM at both timepoints was associated with the majority of significant interactions and involved SNPs of both hydrophobic and hydrophilic SP genes. The rs721917 (C allele) of *SFTPD* is significant by itself and is associated with a decreased risk of PRM. This SNP interacted with other SNPs of the SP genes and was present in the majority of SNP–SNP interactions associated with a decreased risk of PRM. These indicate a protective role of the rs721917 (C allele) of the *SFTPD* gene in the long-term outcomes of ARF survivors; however, the underlying mechanism is unknown. At 12 months, some of the significant interactions are associated with increased or decreased risks of PRM depending on dominant or additive effects of each SNP in that particular interaction in the two-SNP model (Table 5). For example, the interaction between the rs1965708 of the *SFTPA2* and the rs1136450 of the *SFTPA1* is associated with an increased risk of PRM if the interaction type is a1d2, meaning that the rs1965708 and the rs1136450 exhibit additive and dominant effects, respectively. However, the susceptibility to PRM could reverse with reversal of the effects of the involved SNPs, as shown for these two SNPs, if the interaction type, for example, is d1a2. We observed eight such interactions with the same SNPs to associate with either increased or decreased risk of PRM at 12 months based on the effect of SNPs in the given interaction (Table 5).

In the current study, we applied principles of quantitative genetics that help to deconstruct the effects of each SNP on disease susceptibility. The gene dosage is an important factor for normal gene function in health and disease conditions (Veitia and Potier, 2015). Too much or too little of a gene product and their interactions could possibly lead to over-, under-, and/or nonfunction of genes in a disease state (Veitia and Potier, 2015). Furthermore, various studies have shown that the serum concentration and biochemical properties of surfactant proteins are altered in pediatric ARF as assessed by genetic and environmental factors (Sørensen et al., 2006; Dahmer et al., 2020; Saleh et al., 2021). The disease phenotype may change based on a quantitative or qualitative imbalance of a given gene product in a given microenvironment. Together, these observations may explain the change in susceptibility to PRM based on the effect of a particular SNP in a particular interaction. Of note, one intragenic interaction between SNPs (rs1059046 and rs1965707) of the *SFTPA2* is associated with an increased risk of PRM at 12 months. These SNPs, by themselves or in combination, have been shown to associate with an increased risk of severe RSV infection and asthma in children (Lüfgren et al., 2002; Pettigrew et al., 2007; El Saleeby et al., 2010). In the current study, about ∼55% of the patients with PRM had RSV bronchiolitis as an etiology of ARF; hence, our findings are in line with previous observations.

In the three-SNP model, significant intergenic interactions between SNPs of both hydrophobic (*SFTPB*) and hydrophilic SPs (*SFTPA1*, *SFTPA2*, and *SFTPD*) exhibited disease-specific outcomes, meaning the same interaction with similar effects of the involved SNPs decreased the risk of PRM at 6 months but increased the risk of PRM at 12 months (Table 6). Currently, these observations are puzzling and difficult to understand. However, future *in vitro* and/or *in vivo* experiments studying the impact of these gene–gene interactions on the level and properties of SPs in health and disease may help to understand these observations.

The majority of SNPs and their interactions associated with PRM risk at 6 months remained significant at 12 months as well, yet the specific interactions are very distinct from ARF and its short-term outcome in the same cohort (Gandhi et al., 2020a). In fact, the pattern of SNPs and their interactions was unique to each disease population. For example, SNPs of the *SFTPB* and the *SFTPC* by themselves and/or through their interactions were significantly associated with cystic fibrosis (Lin et al., 2018), whereas, SNPs of the *SFTPA1* and *SFTPA2* and their interactions were associated with an increased HP risk in a Mexican population (Gandhi et al., 2021) and RDS in prematurely born neonates (Amatya et al., 2021). The majority of the significant interactions associated with an increased ARF risk involved *SFTPA2* SNPs, whereas the majority of the significant interactions associated with an increased risk of pulmonary dysfunction at discharge involved *SFTPA1 SNP*s in the same dataset (Gandhi et al., 2020a). This is an interesting observation because SP-A2 encoded by SFTPA2 and SP-A1 encoded by *SFTPA1* for the most part exhibit higher activity in innate host defense/inflammatory processes and in surfactant-related functions, respectively (Floros et al., 2021). In the current study, SNPs of the hydrophobic SPs by themselves were associated with an increased risk of PRM, whereas their interactions with the hydrophilic SPs were associated with a decreased risk of PRM at 6 and 12 months. These findings may point at significant roles of a particular set of SNPs and their interactions in ARF and disease progression (short term at 28 days, and long term at 6 and 12 months) in previously healthy children. Based on the odds ratio, of the two- and three-SNP interactions, there is only one for each with an OR of more than 5 that is associated with an increased risk for PRM at 12 months. In the two-SNP model, this interaction is of the a1 effect type, between rs1965707 of the *SFTPA2* × rs1136450 of *SFTPA1*, and in the three-SNP model, the interaction is of the a1d3-effect type, among rs1059046 of the *SFTPA2* × rs1130866 of the *SFTPB* × rs721917 of the *SFTPD.* None of the interactions had ORs of more than 5 in PRM at 6 months. Of interest, infection was the major etiology of ARF in the studied cohort. Considering the vital role of the hydrophilic SPs, particularly SP-A, in innate immunity and host responses of the lung to infection, these findings are not surprising. In the future, if these results are duplicated in a validation cohort, identification of such high-risk interactions could possibly influence clinical decision making for prognostication and counselling of parents of pediatric ARF survivors.

Strengths of this study include 1) the multicenter prospective longitudinal study design enrolling previously healthy children and the well-characterized demographic, illness, and environmental exposure information for the study cohort, and 2) the use of two different statistical approaches adjusting for clinically important variables. Some limitations should be noted for the current study. First, based on the inherent drawbacks of case-control design, the cause–effect explanation is limited. Second, we did not measure the level of SPs in serum or bronchoalvolar lavage fluid; therefore, the impact of these SNPs on SP level is unknown. Third, we only have a moderate sample size and somewhat heterogeneous patient population, despite restricting the study to those with previously healthy lungs. According to the simulation studies of Wang et al. (2010), although this sample size may produce a power of approximately 50%, it can adequately reduce false-positive rates. Thus, while a portion of significant loci remains to be detected using a larger sample size, all significant genetic effects detected in this study deserve a further investigation. More importantly, our study has identified high-order epistatic interactions for persistent respiratory morbidity susceptibility, a genetic phenomenon that has been thought to be important but highly unexplored. The majority of enrolled patients were non-Hispanic Caucasian children; hence, generalization of our findings is limited. In addition, population stratification based on race and ethnicity, and the principal component analysis, were not done, and this omission may have introduced false-positive associations. However, we have adjusted for several variables, including race, to account for difference in allele frequencies among different races. Nonetheless, these associations should be validated and replicated in heterogeneous groups of patients in a sufficiently larger sample size.

In conclusion, we showed, for the first time, the association of SP SNPs with long-term sequelae of ARF survivors in previously healthy children. Our results indicate that both groups of SPs, those involved in normal lung function, and those involved in innate immunity, associate with PRM at 6 and 12 months via complex interactions. The SNP–SNP interaction statistical method helps to identify novel high-order interaction-mediated genotype–phenotype associations not found with the standard univariate/multivariate analyses in the same dataset. The knowledge gained from the current study could be used to develop specific markers to predict long-term sequelae of ARF survivors in previously healthy children, and thus, in the long term, an intervention may be initiated to attenuate the long-term pulmonary sequelae of ARF.

## Data Availability

The original contributions presented in the study are included in the article/Supplementary Material, further inquiries can be directed to the corresponding author.

## References

[B1] Amatya S. Ye M. Yang L. Gandhi C. K. Wu R. Nagourney B. (2021). Single Nucleotide Polymorphisms Interactions of the Surfactant Protein Genes Associated With Respiratory Distress Syndrome Susceptibility in Preterm Infants. Front. Pediatr. 9 (1065), 682160. 10.3389/fped.2021.682160 34671583 PMC8521105

[B2] Amigoni A. Pettenazzo A. Stritoni V. Circelli M. (2017). Surfactants in Acute Respiratory Distress Syndrome in Infants and Children: Past, Present and Future. Clin. Drug Investig. 37 (8), 729–736. 10.1007/s40261-017-0532-1 PMC5509808 28510235

[B3] Chan K. S. Mangione-Smith R. Burwinkle T. M. Rosen M. Varni J. W. (2005). The PedsQL? Med. Care. 43 (3), 256–265. 10.1097/00005650-200503000-00008 15725982

[B4] Cordell H. J. (2009). Detecting Gene-Gene Interactions that Underlie Human Diseases. Nat. Rev. Genet. 10 (6), 392–404. 10.1038/nrg2579 19434077 PMC2872761

[B5] Dahmer M. K. Flori H. Sapru A. Kohne J. Weeks H. M. Curley M. A. Q. (2020). Surfactant Protein D Is Associated With Severe Pediatric ARDS, Prolonged Ventilation, and Death in Children With Acute Respiratory Failure. Chest. 158 (3), 1027–1035. 10.1016/j.chest.2020.03.041 32275979 PMC7478231

[B6] Day N. E. Byar D. P. (1979). Testing Hypotheses in Case-Control Studies-Equivalence of Mantel-Haenszel Statistics and Logit Score Tests. Biometrics. 35, 623–630. 10.2307/2530253 497345

[B7] Depicolzuane L. Phelps D. S. Floros J. (2021). Surfactant Protein-A Function: Knowledge Gained From SP-A Knockout Mice. Front. Pediatr. 9, 799693. 10.3389/fped.2021.799693 35071140 PMC8777267

[B8] DiAngelo S. Lin Z. Wang G. Phillips S. Ramet M. Luo J. (1999). Novel, Non-radioactive, Simple and Multiplex PCR-cRFLP Methods for Genotyping Human SP-A and SP-D Marker Alleles. Dis. Markers. 15 (4), 269–281. 10.1155/1999/961430 10689550 PMC3851098

[B9] El Saleeby C. M. Li R. Somes G. W. Dahmer M. K. Quasney M. W. DeVincenzo J. P. (2010). Surfactant Protein A2 Polymorphisms and Disease Severity in a Respiratory Syncytial Virus-Infected Population. J. Pediatr. 156 (3), 409–414. 10.1016/j.jpeds.2009.09.043 19914637

[B10] Floros J. Fan R. Diangelo S. Guo X. Wert J. Luo J. (2001). Surfactant Protein (SP) B Associations and Interactions with SP-A in white and Black Subjects with Respiratory Distress Syndrome. Pediatr. Int. 43 (6), 567–576. 10.1046/j.1442-200x.2001.01474.x 11737731 PMC2907917

[B11] Floros J. Lin H. M. García A. Salazar M. A. Guo X. DiAngelo S. (2000). Surfactant Protein Genetic Marker Alleles Identify a Subgroup of Tuberculosis in a Mexican Population. J. Infect. Dis. 182 (5), 1473–1478. 10.1086/315866 11023470

[B12] Floros J. Thorenoor N. Tsotakos N. Phelps D. S. (2021). Human Surfactant Protein SP-A1 and SP-A2 Variants Differentially Affect the Alveolar Microenvironment, Surfactant Structure, Regulation and Function of the Alveolar Macrophage, and Animal and Human Survival under Various Conditions. Front. Immunol. 12 (2889), 681639. 10.3389/fimmu.2021.681639 34484180 PMC8415824

[B13] Floros J. Tsotakos N. (2021). Differential Regulation of Human Surfactant Protein A Genes, SFTPA1 and SFTPA2, and Their Corresponding Variants. Front. Immunol. 12, 766719. 10.3389/fimmu.2021.766719 34917085 PMC8669794

[B14] Gandhi C. K. Chen C. Amatya S. Yang L. Fu C. Zhou S. (2021). SNP and Haplotype Interaction Models Reveal Association of Surfactant Protein Gene Polymorphisms With Hypersensitivity Pneumonitis of Mexican Population. Front. Med. 7, 588404. 10.3389/fmed.2020.588404 PMC7813780 33469544

[B15] Gandhi C. K. Chen C. Wu R. Yang L. Thorenoor N. Thomas N. J. (2020a). Association of SNP-SNP Interactions of Surfactant Protein Genes with Pediatric Acute Respiratory Failure. J Clin Med. 9 (4), 1183. 10.3390/jcm9041183 PMC7231046 32326132

[B16] Gandhi C. K. Mikerov A. N. Durrani F. Umstead T. M. Hu S. Wang G. (2020b). Impact of Ozone, Sex, and Gonadal Hormones on Bronchoalveolar Lavage Characteristics and Survival in SP-A KO Mice Infected with *Klebsiella pneumoniae* . Microorganisms. 8 (9), 1354. 10.3390/microorganisms8091354 PMC7563396 32899781

[B17] García-Laorden M. I. Rodríguez de Castro F. Solé-Violán J. Rajas O. Blanquer J. Borderías L. (2011). Influence of Genetic Variability at the Surfactant Proteins A and D in Community-Acquired Pneumonia: a Prospective, Observational, Genetic Study. Crit. Care. 15 (1), R57. 10.1186/cc10030 21310059 PMC3221990

[B18] Glasser S. W. Burhans M. S. Korfhagen T. R. Na C.-L. Sly P. D. Ross G. F. (2001). Altered Stability of Pulmonary Surfactant in SP-C-Deficient Mice. Proc. Natl. Acad. Sci. 98 (11), 6366–6371. 10.1073/pnas.101500298 11344267 PMC33474

[B19] Greenley R. N. Josie K. L. Drotar D. (2008). Self-reported Quality of Life Among Inner-City Youth with Asthma: an Empirical Examination of the PedsQL 3.0 Asthma Module. Ann. Allergy Asthma Immunol. 100 (2), 106–111. 10.1016/s1081-1206(10)60418-8 18320911

[B20] Heidinger K. König I. R. Bohnert A. Kleinsteiber A. Hilgendorff A. Gortner L. (2005). Polymorphisms in the Human Surfactant Protein-D (SFTPD) Gene: strong Evidence that Serum Levels of Surfactant Protein-D (SP-D) Are Genetically Influenced. Immunogenetics. 57 (1), 1–7. 10.1007/s00251-005-0775-5 15700120 PMC7100655

[B21] Hochberg Y. B. a. Y. (1995). Controlling the False Discovery Rate: A Practical and Powerful Approach to Multiple Testing. J. R. Stat. Soc. Ser. B (Methodological). 57 (1), 289–300. 10.1111/j.2517-6161.1995.tb02031.x

[B22] Hope A. C. A. (1968). A Simplified Monte Carlo Significance Test Procedure. J. R. Stat. Soc. Ser. B (Methodological). 30, 582–598. 10.1111/j.2517-6161.1968.tb00759.x

[B23] Ibiebele I. Algert C. S. Bowen J. R. Roberts C. L. (2018). Pediatric Admissions that Include Intensive Care: a Population-Based Study. BMC Health Serv. Res. 18 (1), 264. 10.1186/s12913-018-3041-x 29631570 PMC5892018

[B24] Improta R. Benzi C. Barone V. (2001). Understanding the Role of Stereoelectronic Effects in Determining Collagen Stability. 1. A Quantum Mechanical Study of Proline, Hydroxyproline, and Fluoroproline Dipeptide Analogues in Aqueous Solution. J. Am. Chem. Soc. 123 (50), 12568–12577. 10.1021/ja010599i 11741421

[B25] Kala P. Have T. T. Nielsen H. Dunn M. Floros J. (1998). Association of Pulmonary Surfactant Protein A (SP-A) Gene and Respiratory Distress Syndrome: Interaction with SP-B. Pediatr. Res. 43 (2), 169–177. 10.1203/00006450-199802000-00003 9475280

[B26] Keim G. Watson R. S. Thomas N. J. Yehya N. (2018). New Morbidity and Discharge Disposition of Pediatric Acute Respiratory Distress Syndrome Survivors. Crit. Care Med. 46 (11), 1731–1738. 10.1097/ccm.0000000000003341 30024428 PMC6185805

[B27] Keim G. Yehya N. Spear D. Hall M. W. Loftis L. L. Alten J. A. (2020). Development of Persistent Respiratory Morbidity in Previously Healthy Children After Acute Respiratory Failure. Crit. Care Med. 48 (8), 1120–1128. 10.1097/ccm.0000000000004380 32697481 PMC7490803

[B28] Khemani R. G. Smith L. Lopez-Fernandez Y. M. Kwok J. Morzov R. Klein M. J. (2019). Paediatric Acute Respiratory Distress Syndrome Incidence and Epidemiology (PARDIE): an International, Observational Study. Lancet Respir. Med. 7 (2), 115–128. 10.1016/s2213-2600(18)30344-8 30361119 PMC7045907

[B29] Kishore U. Greenhough T. J. Waters P. Shrive A. K. Ghai R. Kamran M. F. (2006). Surfactant Proteins SP-A and SP-D: Structure, Function and Receptors. Mol. Immunol. 43 (9), 1293–1315. 10.1016/j.molimm.2005.08.004 16213021

[B30] Lahti M. Löfgren J. Marttila R. Renko M. Klaavuniemi T. Haataja R. (2002). Surfactant Protein D Gene Polymorphism Associated with Severe Respiratory Syncytial Virus Infection. Pediatr. Res. 51 (6), 696–699. 10.1203/00006450-200206000-00006 12032263

[B31] Lin Z. deMello D. Batanian J. Khammash H. DiAngelo S. Luo J. (2000a). Aberrant SP-B mRNA in Lung Tissue of Patients with Congenital Alveolar Proteinosis (CAP). Clin. Genet. 57 (5), 359–369. 10.1034/j.1399-0004.2000.570506.x 10852370

[B32] Lin Z. Pearson C. Chinchilli V. Pietschmann S. Luo J. Pison U. (2000b). Polymorphisms of humanSP-A,SP-B, andSP-Dgenes: Association ofSP-BThr131Ile with ARDS. Clin. Genet. 58 (3), 181–191. 10.1034/j.1399-0004.2000.580305.x 11076040

[B33] Lin Z. Thorenoor N. Wu R. DiAngelo S. L. Ye M. Thomas N. J. (2018). Genetic Association of Pulmonary Surfactant Protein Genes, SFTPA1, SFTPA2, SFTPB, SFTPC, and SFTPD With Cystic Fibrosis. Front. Immunol. 9, 2256. 10.3389/fimmu.2018.02256 30333828 PMC6175982

[B34] Lüfgren J. Rämet M. Renko M. Marttila R. Hallman M. (2002). Association between Surfactant Protein A Gene Locus and Severe Respiratory Syncytial Virus Infection in Infants. J. Infect. Dis. 185 (3), 283–289. 10.1086/338473 11807709

[B35] Madan T. Saxena S. Murthy K. J. Muralidhar K. Sarma P. U. (2002). Association of Polymorphisms in the Collagen Region of Human SP-A1 and SP-A2 Genes with Pulmonary Tuberculosis in Indian Population. Clin. Chem. Lab Med. 40 (10), 1002–1008. 10.1515/CCLM.2002.174 12476938

[B36] Marchini J. Donnelly P. Cardon L. R. (2005). Genome-wide Strategies for Detecting Multiple Loci that Influence Complex Diseases. Nat. Genet. 37 (4), 413–417. 10.1038/ng1537 15793588

[B37] Matthay M. A. McAuley D. F. Ware L. B. (2017). Clinical Trials in Acute Respiratory Distress Syndrome: Challenges and Opportunities. Lancet Respir. Med. 5 (6), 524–534. 10.1016/s2213-2600(17)30188-1 28664851

[B38] Nogee L. M. Wert S. E. Proffit S. A. Hull W. M. Whitsett J. A. (2000). Allelic Heterogeneity in Hereditary Surfactant Protein B (SP-B) Deficiency. Am. J. Respir. Crit. Care Med. 161 (3 Pt 1), 973–981. 10.1164/ajrccm.161.3.9903153 10712351

[B39] Pettigrew M. M. Gent J. F. Zhu Y. Triche E. W. Belanger K. D. Holford T. R. (2007). Respiratory Symptoms Among Infants at Risk for Asthma: Association with Surfactant Protein A Haplotypes. BMC Med. Genet. 8, 15. 10.1186/1471-2350-8-15 17407567 PMC1852548

[B40] Pollack M. M. Holubkov R. Glass P. Dean J. M. Meert K. L. Zimmerman J. (2009). Functional Status Scale: New Pediatric Outcome Measure. Pediatrics. 124 (1), e18–e28. 10.1542/peds.2008-1987 19564265 PMC3191069

[B41] Puthothu B. Krueger M. Heinze J. Forster J. Heinzmann A. (2006). Haplotypes of Surfactant Protein C Are Associated with Common Paediatric Lung Diseases. Pediatr. Allergy Immunol. 17 (8), 572–577. 10.1111/j.1399-3038.2006.00467.x 17121584

[B42] Quasney M. W. Waterer G. W. Dahmer M. K. Kron G. K. Zhang Q. Kessler L. A. (2004). Association between Surfactant Protein B + 1580 Polymorphism and the Risk of Respiratory Failure in Adults with Community-Acquired Pneumonia. Crit. Care Med. 32 (5), 1115–1119. 10.1097/01.ccm.0000124872.55243.5a 15190959

[B43] Rämet M. Haataja R. Marttila R. Floros J. Hallman M. (2000). Association between the Surfactant Protein A (SP-A) Gene Locus and Respiratory-Distress Syndrome in the Finnish Population. Am. J. Hum. Genet. 66 (5), 1569–1579. 10.1086/302906 10762543 PMC1378016

[B44] Saleh N. Y. Ibrahem R. A. L. Saleh A. A. h. Soliman S. E. s. Mahmoud A. A. S. (2021). Surfactant Protein D: a Predictor for Severity of Community-Acquired Pneumonia in Children. Pediatr. Res. 91, 665–671. 10.1038/s41390-021-01492-9 33790414 PMC8010482

[B45] Saxena S. Madan T. Shah A. Muralidhar K. Sarma P. U. (2003). Association of Polymorphisms in the Collagen Region of SP-A2 with Increased Levels of Total IgE Antibodies and Eosinophilia in Patients with Allergic Bronchopulmonary Aspergillosis. J. Allergy Clin. Immunol. 111 (5), 1001–1007. 10.1067/mai.2003.1395 12743564

[B46] Seid M. Limbers C. A. Driscoll K. A. Opipari-Arrigan L. A. Gelhard L. R. Varni J. W. (2010). Reliability, Validity, and Responsiveness of the Pediatric Quality of Life Inventory (PedsQL) Generic Core Scales and Asthma Symptoms Scale in Vulnerable Children with Asthma. J. Asthma. 47 (2), 170–177. 10.3109/02770900903533966 20170325

[B47] Seifart C. Plagens A. Brödje D. Müller B. von Wichert P. Floros J. (2002). Surfactant Protein B Intron 4 Variation in German Patients with COPD and Acute Respiratory Failure. Dis. Markers. 18 (3), 129–136. 10.1155/2002/194075 12515908 PMC3851100

[B48] Selman M. Lin H.-M. Montao M. Jenkins A. L. Estrada A. Lin Z. (2003). Surfactant proteinA and B Genetic Variants Predispose to Idiopathic Pulmonary Fibrosis. Hum. Genet. 113 (6), 542–550. 10.1007/s00439-003-1015-4 13680361

[B49] Serrano A. G. Pérez-Gil J. (2006). Protein-lipid Interactions and Surface Activity in the Pulmonary Surfactant System. Chem. Phys. Lipids. 141 (1-2), 105–118. 10.1016/j.chemphyslip.2006.02.017 16600200

[B50] Sherry S. T. Ward M. H. Kholodov M. Baker J. Phan L. Smigielski E. M. (2001). dbSNP: the NCBI Database of Genetic Variation. Nucleic Acids Res. 29 (1), 308–311. 10.1093/nar/29.1.308 11125122 PMC29783

[B51] Silveyra P. Floros J. (2012). Genetic Variant Associations of Human SP-A and SP-D with Acute and Chronic Lung Injury. Front. Biosci. 17, 407–429. 10.2741/3935 PMC3635489 22201752

[B52] Sørensen G. L. Hjelmborg J. v. B. Kyvik K. O. Fenger M. Høj A. Bendixen C. (2006). Genetic and Environmental Influences of Surfactant Protein D Serum Levels. Am. J. Physiology-Lung Cell Mol. Physiol. 290 (5), L1010–L1017. 10.1152/ajplung.00487.2005 16361352

[B53] Taponen S. Huusko J. M. Petäjä-Repo U. E. Paananen R. Guttentag S. H. Hallman M. (2013). Allele-specific N-Glycosylation Delays Human Surfactant Protein B Secretion *In Vitro* and Associates with Decreased Protein Levels *In Vivo* . Pediatr. Res. 74 (6), 646–651. 10.1038/pr.2013.151 24002332

[B54] Thomas N. J. DiAngelo S. Hess J. C. Fan R. Ball M. W. Geskey J. M. (2009). Transmission of Surfactant Protein Variants and Haplotypes in Children Hospitalized with Respiratory Syncytial Virus. Pediatr. Res. 66 (1), 70–73. 10.1203/PDR.0b013e3181a1d768 19287351 PMC2710771

[B55] Thorenoor N. Kawasawa Y. I. Gandhi C. K. Floros J. (2020). Sex-Specific Regulation of Gene Expression Networks by Surfactant Protein A (SP-A) Variants in Alveolar Macrophages in Response to *Klebsiella pneumoniae* . Front. Immunol. 11, 1290. 10.3389/fimmu.2020.01290 32670284 PMC7326812

[B56] Thorenoor N. Kawasawa Y. I. Gandhi C. K. Zhang X. Floros J. (2019). Differential Impact of Co-expressed SP-A1/sp-A2 Protein on AM miRNome; Sex Differences. Front. Immunol. 10, 1960. 10.3389/fimmu.2019.01960 PMC6707024 31475015

[B57] Veitia R. A. Potier M. C. (2015). Gene Dosage Imbalances: Action, Reaction, and Models. Trends Biochem. Sci. 40 (6), 309–317. 10.1016/j.tibs.2015.03.011 25937627

[B58] Wang G. Christensen N. D. Wigdahl B. Guttentag S. H. Floros J. (2003). Differences in N-Linked Glycosylation between Human Surfactant Protein-B Variants of the C or T Allele at the Single-Nucleotide Polymorphism at Position 1580: Implications for Disease. Biochem. J. 369 (Pt 1), 179–184. 10.1042/bj20021376 12356334 PMC1223069

[B59] Wang Z. Liu T. Lin Z. Hegarty J. Koltun W. A. Wu R. (2010). A General Model for Multilocus Epistatic Interactions in Case-Control Studies. PLoS One. 5 (8), e11384. 10.1371/journal.pone.0011384 20814428 PMC2909900

[B60] Wert S. E. Whitsett J. A. Nogee L. M. (2009). Genetic Disorders of Surfactant Dysfunction. Pediatr. Dev. Pathol. 12 (4), 253–274. 10.2350/09-01-0586.1 19220077 PMC2987676

[B61] Wright J. R. (2005). Immunoregulatory Functions of Surfactant Proteins. Nat. Rev. Immunol. 5 (1), 58–68. 10.1038/nri1528 15630429

[B62] Xu H. N. Lin Z. Gandhi C. K. Amatya S. Wang Y. Li L. Z. (2020). Sex and SP-A2 Dependent NAD(H) Redox Alterations in Mouse Alveolar Macrophages in Response to Ozone Exposure: Potential Implications for COVID-19. Antioxidants. 9 (10), 915. 10.3390/antiox9100915 PMC7601279 32992843

[B63] Xu Y. Ge L. Abdel-Razek O. Jain S. Liu Z. Hong Y. (2016). Differential Susceptibility of Human Sp-B Genetic Variants on Lung Injury Caused by Bacterial Pneumonia and the Effect of a Chemically Modified Curcumin. Shock (Augusta, Ga.). 45 (4), 375–384. 10.1097/shk.0000000000000535 PMC4792714 26863117

[B64] Yang F. Zhang J. Yang Y. Ruan F. Chen X. Guo J. (2019). Regulatory Roles of Human Surfactant Protein B Variants on Genetic Susceptibility to Pseudomonas Aeruginosa Pneumonia-Induced Sepsis. Shock. 54, 507–519. 10.1097/shk.0000000000001494 PMC10336628 31851120

[B65] Yehya N. Thomas N. J. (2016). Relevant Outcomes in Pediatric Acute Respiratory Distress Syndrome Studies. Front. Pediatr. 4, 51. 10.3389/fped.2016.00051 27242980 PMC4865511

