# Supplementary material for: SNP–SNP Interactions of Surfactant Protein Genes in Persistent Respiratory Morbidity Susceptibility in Previously Healthy Children
Source: Front Genet. 2022 Mar 24;13:815727. doi: 10.3389/fgene.2022.815727 (PMC8989419; doi:10.3389/fgene.2022.815727)
Supplement: Supplementary file 1 [file DataSheet1.docx]

**Supplementary table 1** **Details of the studied SNPs**

| **Gene** | **SNP ID** | **Nucleotide Change** | **Other used name** |
| --- | --- | --- | --- |
| *SFTPA1* | rs1059047 | T/C | aa19 Ala/Val |
|  | rs1136450 | C/G | aa50 Leu/Val |
|  | rs1136451 | A/G | aa62 Pro/Pro |
|  | rs1059057 | A/G | aa133 Thr/Thr |
|  | rs4253527 | C/T | aa219 Arg/Trp |
| *SFTPA2* | rs1059046 | G/C | aa9 Asn/Thr |
|  | rs17886395 | C/G | aa91 Pro/Ala |
|  | rs1965707 | G/A | aa140 Ser/Ser |
|  | rs1965708 | G/T | aa223 Gln/Lys |
| *SFTPB* | rs2077079* | T/G | CA-18, CA1022 |
|  | rs3024798* | G/T | CA1013, CA2052 |
|  | rs1130866 | G/A | TC1580, TC2619  aa131 Ile/Thr |
|  | rs7316* | T/C | AG9306, AG10345 |
| *SFTPC* | rs4715 | C/A | aa138 Asn/Thr |
|  | rs1124 | G/A | aa186 Asn/Ser |
| *SFTPD* | rs721917 | A/G | aa11 Met/Thr |
|  | rs2243639 | T/C | aa160 Thr/Ala |

Numbering of amino acids in *SFTPA1* and *SFTPA2*, is that of the precursor molecule i.e. includes the signal peptide. Numbering of amino acids in *SFTPB*, *SFTPC* and *SFTPD* is based on the mature/precursor protein minus the signal peptide.

All the studied SNP are located within the exons, except the three *SFTPB* marked with *. The SFTPB a) rs2077079 is located 10 nt downstream of TATAA box, 5’ regulatory region; b) rs3024798 is located in the intron at the splice sequence of intron 2-exon 3; and c) rs7316 is located in the 3’UTR 4 nucleotides upstream of the TAATAA polyadenylation signal.

**Supplementary Table 2 Genotype frequencies in population of persistent respiratory morbidity at 6 months**

| **SNPs** | **Race** | **Group** | **Genotype 1** | **Genotype 2** | **Genotype 3** |
| --- | --- | --- | --- | --- | --- |
| rs1059046 | Hispanic |  | AA | AC | CC |
|  |  | Case | 3 (0.2) | 8 (0.53) | 4 (0.27) |
|  |  | Control | 4 (0.31) | 4 (0.31) | 5 (0.38) |
|  | White |  | AA | AC | CC |
|  |  | Case | 18 (0.34) | 29 (0.55) | 6 (0.11) |
|  |  | Control | 14 (0.4) | 13 (0.37) | 8 (0.23) |
|  | Asian or Pacific Islander |  | AA | AC | CC |
|  |  | Case | 1 (0.25) | 1 (0.25) | 2 (0.5) |
|  |  | Control | 0 (0) | 1 (0.5) | 1 (0.5) |
|  | Black, not of Hispanic origin |  | AA | AC | CC |
|  |  | Case | 5 (0.42) | 4 (0.33) | 3 (0.25) |
|  |  | Control | 1 (0.09) | 6 (0.55) | 4 (0.36) |
|  | Other or Unknown (mixed parents) | | AA | AC | CC |
|  |  | Case | 2 (0.4) | 0 (0) | 3 (0.6) |
|  |  | Control | 3 (0.75) | 1 (0.25) | 0 (0) |
| rs17886395 | Hispanic |  | CC | CG | GG |
|  |  | Case | 1 (0.07) | 5 (0.33) | 9 (0.6) |
|  |  | Control | 1 (0.08) | 3 (0.23) | 9 (0.69) |
|  | White |  | CC | CG | GG |
|  |  | Case | 1 (0.02) | 15 (0.28) | 37 (0.7) |
|  |  | Control | 2 (0.06) | 15 (0.43) | 18 (0.51) |
|  | Asian or Pacific Islander |  | CC | CG | GG |
|  |  | Case | 0 (0) | 2 (0.5) | 2 (0.5) |
|  |  | Control | 0 (0) | 2 (1) | 0 (0) |
|  | Black, not of Hispanic origin |  | CC | CG | GG |
|  |  | Case | 0 (0) | 4 (0.33) | 8 (0.67) |
|  |  | Control | 0 (0) | 5 (0.45) | 6 (0.55) |
|  | Other or Unknown (mixed parents) | | CC | CG | GG |
|  |  | Case | 0 (0) | 3 (0.6) | 2 (0.4) |
|  |  | Control | 0 (0) | 0 (0) | 4 (1) |
| rs1965707 | Hispanic |  | CC | CT | TT |
|  |  | Case | 5 (0.33) | 8 (0.53) | 2 (0.13) |
|  |  | Control | 8 (0.62) | 3 (0.23) | 2 (0.15) |
|  | White |  | CC | CT | TT |
|  |  | Case | 27 (0.51) | 22 (0.42) | 4 (0.08) |
|  |  | Control | 17 (0.49) | 16 (0.46) | 2 (0.06) |
|  | Asian or Pacific Islander |  | CC | CT | TT |
|  |  | Case | 2 (0.5) | 2 (0.5) | 0 (0) |
|  |  | Control | 1 (0.5) | 0 (0) | 1 (0.5) |
|  | Black, not of Hispanic origin |  | CC | CT | TT |
|  |  | Case | 9 (0.75) | 2 (0.17) | 1 (0.08) |
|  |  | Control | 6 (0.55) | 3 (0.27) | 2 (0.18) |
|  | Other or Unknown (mixed parents) | | CC | CT | TT |
|  |  | Case | 3 (0.6) | 1 (0.2) | 1 (0.2) |
|  |  | Control | 2 (0.5) | 2 (0.5) | 0 (0) |
| rs1965708 | Hispanic |  | AA | AC | CC |
|  |  | Case | 1 (0.07) | 6 (0.4) | 8 (0.53) |
|  |  | Control | 1 (0.08) | 3 (0.23) | 9 (0.69) |
|  | White |  | AA | AC | CC |
|  |  | Case | 2 (0.04) | 18 (0.34) | 33 (0.62) |
|  |  | Control | 0 (0) | 10 (0.29) | 25 (0.71) |
|  | Asian or Pacific Islander |  | AA | AC | CC |
|  |  | Case | 0 (0) | 1 (0.25) | 3 (0.75) |
|  |  | Control | 0 (0) | 1 (0.5) | 1 (0.5) |
|  | Black, not of Hispanic origin |  | AA | AC | CC |
|  |  | Case | 1 (0.08) | 2 (0.17) | 9 (0.75) |
|  |  | Control | 1 (0.09) | 5 (0.45) | 5 (0.45) |
|  | Other or Unknown (mixed parents) | | AA | AC | CC |
|  |  | Case | 0 (0) | 2 (0.4) | 3 (0.6) |
|  |  | Control | 0 (0) | 2 (0.5) | 2 (0.5) |
| rs1059047 | Hispanic |  | CC | CT | TT |
|  |  | Case | 0 (0) | 4 (0.29) | 10 (0.71) |
|  |  | Control | 1 (0.08) | 3 (0.25) | 8 (0.67) |
|  | White |  | CC | CT | TT |
|  |  | Case | 0 (0) | 8 (0.16) | 42 (0.84) |
|  |  | Control | 0 (0) | 7 (0.21) | 26 (0.79) |
|  | Asian or Pacific Islander |  | CC | CT | TT |
|  |  | Case | 0 (0) | 1 (0.25) | 3 (0.75) |
|  |  | Control | 0 (0) | 1 (0.5) | 1 (0.5) |
|  | Black, not of Hispanic origin |  | CC | CT | TT |
|  |  | Case | 0 (0) | 3 (0.27) | 8 (0.73) |
|  |  | Control | 0 (0) | 0 (0) | 10 (1) |
|  | Other or Unknown (mixed parents) | | CC | CT | TT |
|  |  | Case | 0 (0) | 2 (0.4) | 3 (0.6) |
|  |  | Control | 0 (0) | 0 (0) | 4 (1) |
| rs1136450 | Hispanic |  | CC | CG | GG |
|  |  | Case | 4 (0.29) | 9 (0.64) | 1 (0.07) |
|  |  | Control | 5 (0.42) | 5 (0.42) | 2 (0.17) |
|  | White |  | CC | CG | GG |
|  |  | Case | 7 (0.14) | 24 (0.48) | 19 (0.38) |
|  |  | Control | 7 (0.21) | 14 (0.42) | 12 (0.36) |
|  | Asian or Pacific Islander |  | CC | CG | GG |
|  |  | Case | 2 (0.5) | 1 (0.25) | 1 (0.25) |
|  |  | Control | 2 (1) | 0 (0) | 0 (0) |
|  | Black, not of Hispanic origin |  | CC | CG | GG |
|  |  | Case | 2 (0.18) | 4 (0.36) | 5 (0.45) |
|  |  | Control | 2 (0.2) | 6 (0.6) | 2 (0.2) |
|  | Other or Unknown (mixed parents) | | CC | CG | GG |
|  |  | Case | 3 (0.6) | 0 (0) | 2 (0.4) |
|  |  | Control | 0 (0) | 2 (0.5) | 2 (0.5) |
| rs1136451 | Hispanic |  | AA | AG | GG |
|  |  | Case | 7 (0.5) | 6 (0.43) | 1 (0.07) |
|  |  | Control | 5 (0.42) | 4 (0.33) | 3 (0.25) |
|  | White |  | AA | AG | GG |
|  |  | Case | 35 (0.7) | 13 (0.26) | 2 (0.04) |
|  |  | Control | 20 (0.61) | 11 (0.33) | 2 (0.06) |
|  | Asian or Pacific Islander |  | AA | AG | GG |
|  |  | Case | 2 (0.5) | 2 (0.5) | 0 (0) |
|  |  | Control | 0 (0) | 2 (1) | 0 (0) |
|  | Black, not of Hispanic origin |  | AA | AG | GG |
|  |  | Case | 8 (0.73) | 2 (0.18) | 1 (0.09) |
|  |  | Control | 8 (0.8) | 2 (0.2) | 0 (0) |
|  | Other or Unknown (mixed parents) | | AA | AG | GG |
|  |  | Case | 2 (0.4) | 3 (0.6) | 0 (0) |
|  |  | Control | 4 (1) | 0 (0) | 0 (0) |
| rs1059057 | Hispanic |  | AA | AG | GG |
|  |  | Case | 10 (0.71) | 4 (0.29) | 0 (0) |
|  |  | Control | 9 (0.75) | 2 (0.17) | 1 (0.08) |
|  | White |  | AA | AG | GG |
|  |  | Case | 42 (0.84) | 8 (0.16) | 0 (0) |
|  |  | Control | 26 (0.79) | 7 (0.21) | 0 (0) |
|  | Asian or Pacific Islander |  | AA | AG | GG |
|  |  | Case | 3 (0.75) | 1 (0.25) | 0 (0) |
|  |  | Control | 1 (0.5) | 1 (0.5) | 0 (0) |
|  | Black, not of Hispanic origin |  | AA | AG | GG |
|  |  | Case | 8 (0.73) | 3 (0.27) | 0 (0) |
|  |  | Control | 10 (1) | 0 (0) | 0 (0) |
|  | Other or Unknown (mixed parents) | | AA | AG | GG |
|  |  | Case | 3 (0.6) | 2 (0.4) | 0 (0) |
|  |  | Control | 4 (1) | 0 (0) | 0 (0) |
| rs4253527 | Hispanic |  | CC | CT | TT |
|  |  | Case | 10 (0.71) | 4 (0.29) | 0 (0) |
|  |  | Control | 7 (0.58) | 4 (0.33) | 1 (0.08) |
|  | White |  | CC | CT | TT |
|  |  | Case | 40 (0.8) | 10 (0.2) | 0 (0) |
|  |  | Control | 25 (0.76) | 8 (0.24) | 0 (0) |
|  | Asian or Pacific Islander |  | CC | CT | TT |
|  |  | Case | 3 (0.75) | 1 (0.25) | 0 (0) |
|  |  | Control | 1 (0.5) | 1 (0.5) | 0 (0) |
|  | Black, not of Hispanic origin |  | CC | CT | TT |
|  |  | Case | 10 (0.91) | 1 (0.09) | 0 (0) |
|  |  | Control | 8 (0.8) | 2 (0.2) | 0 (0) |
|  | Other or Unknown (mixed parents) | | CC | CT | TT |
|  |  | Case | 4 (0.8) | 1 (0.2) | 0 (0) |
|  |  | Control | 4 (1) | 0 (0) | 0 (0) |
| rs1130866 | Hispanic |  | CC | CT | TT |
|  |  | Case | 3 (0.2) | 8 (0.53) | 4 (0.27) |
|  |  | Control | 7 (0.54) | 5 (0.38) | 1 (0.08) |
|  | White |  | CC | CT | TT |
|  |  | Case | 9 (0.17) | 25 (0.48) | 18 (0.35) |
|  |  | Control | 8 (0.22) | 23 (0.64) | 5 (0.14) |
|  | Asian or Pacific Islander |  | CC | CT | TT |
|  |  | Case | 0 (0) | 3 (0.75) | 1 (0.25) |
|  |  | Control | 0 (0) | 2 (1) | 0 (0) |
|  | Black, not of Hispanic origin |  | CC | CT | TT |
|  |  | Case | 2 (0.17) | 7 (0.58) | 3 (0.25) |
|  |  | Control | 1 (0.09) | 1 (0.09) | 9 (0.82) |
|  | Other or Unknown (mixed parents) | | CC | CT | TT |
|  |  | Case | 1 (0.2) | 3 (0.6) | 1 (0.2) |
|  |  | Control | 1 (0.25) | 2 (0.5) | 1 (0.25) |
| rs4715 | Hispanic |  | AA | AC | CC |
|  |  | Case | 1 (0.07) | 3 (0.2) | 11 (0.73) |
|  |  | Control | 0 (0) | 8 (0.62) | 5 (0.38) |
|  | White |  | AA | AC | CC |
|  |  | Case | 4 (0.08) | 16 (0.3) | 33 (0.62) |
|  |  | Control | 1 (0.03) | 13 (0.37) | 21 (0.6) |
|  | Asian or Pacific Islander |  | AA | AC | CC |
|  |  | Case | 0 (0) | 2 (0.5) | 2 (0.5) |
|  |  | Control | 0 (0) | 0 (0) | 2 (1) |
|  | Black, not of Hispanic origin |  | AA | AC | CC |
|  |  | Case | 2 (0.17) | 4 (0.33) | 6 (0.5) |
|  |  | Control | 0 (0) | 0 (0) | 11 (1) |
|  | Other or Unknown (mixed parents) | | AA | AC | CC |
|  |  | Case | 0 (0) | 3 (0.6) | 2 (0.4) |
|  |  | Control | 0 (0) | 2 (0.5) | 2 (0.5) |
| rs1124 | Hispanic |  | AA | AG | GG |
|  |  | Case | 1 (0.07) | 7 (0.47) | 7 (0.47) |
|  |  | Control | 0 (0) | 8 (0.62) | 5 (0.38) |
|  | White |  | AA | AG | GG |
|  |  | Case | 7 (0.13) | 21 (0.4) | 25 (0.47) |
|  |  | Control | 1 (0.03) | 16 (0.44) | 19 (0.53) |
|  | Asian or Pacific Islander |  | AA | AG | GG |
|  |  | Case | 0 (0) | 2 (0.5) | 2 (0.5) |
|  |  | Control | 0 (0) | 0 (0) | 2 (1) |
|  | Black, not of Hispanic origin |  | AA | AG | GG |
|  |  | Case | 3 (0.25) | 4 (0.33) | 5 (0.42) |
|  |  | Control | 0 (0) | 2 (0.18) | 9 (0.82) |
|  | Other or Unknown (mixed parents) | | AA | AG | GG |
|  |  | Case | 0 (0) | 3 (0.6) | 2 (0.4) |
|  |  | Control | 1 (0.25) | 2 (0.5) | 1 (0.25) |
| rs721917 | Hispanic |  | CC | CT | TT |
|  |  | Case | 3 (0.2) | 10 (0.67) | 2 (0.13) |
|  |  | Control | 5 (0.38) | 5 (0.38) | 3 (0.23) |
|  | White |  | CC | CT | TT |
|  |  | Case | 8 (0.15) | 32 (0.6) | 13 (0.25) |
|  |  | Control | 6 (0.17) | 18 (0.5) | 12 (0.33) |
|  | Asian or Pacific Islander |  | CC | CT | TT |
|  |  | Case | 1 (0.25) | 1 (0.25) | 2 (0.5) |
|  |  | Control | 1 (0.5) | 1 (0.5) | 0 (0) |
|  | Black, not of Hispanic origin |  | CC | CT | TT |
|  |  | Case | 1 (0.08) | 10 (0.83) | 1 (0.08) |
|  |  | Control | 2 (0.18) | 4 (0.36) | 5 (0.45) |
|  | Other or Unknown (mixed parents) | | CC | CT | TT |
|  |  | Case | 2 (0.4) | 2 (0.4) | 1 (0.2) |
|  |  | Control | 0 (0) | 2 (0.5) | 2 (0.5) |
| rs2243639 | Hispanic |  | AA | AG | GG |
|  |  | Case | 1 (0.07) | 5 (0.33) | 9 (0.6) |
|  |  | Control | 2 (0.15) | 3 (0.23) | 8 (0.62) |
|  | White |  | AA | AG | GG |
|  |  | Case | 4 (0.08) | 29 (0.55) | 20 (0.38) |
|  |  | Control | 7 (0.2) | 16 (0.46) | 12 (0.34) |
|  | Asian or Pacific Islander |  | AA | AG | GG |
|  |  | Case | 0 (0) | 1 (0.25) | 3 (0.75) |
|  |  | Control | 0 (0) | 0 (0) | 2 (1) |
|  | Black, not of Hispanic origin |  | AA | AG | GG |
|  |  | Case | 2 (0.17) | 7 (0.58) | 3 (0.25) |
|  |  | Control | 0 (0) | 3 (0.27) | 8 (0.73) |
|  | Other or Unknown (mixed parents) | | AA | AG | GG |
|  |  | Case | 0 (0) | 1 (0.2) | 4 (0.8) |
|  |  | Control | 1 (0.25) | 2 (0.5) | 1 (0.25) |

**Supplementary Table 3 Genotype frequencies in population of persistent respiratory morbidity at 12 months**

| **SNP** | **Race** | **Group** | **Genotype 1** | **Genotype 2** | **Genotype 3** |
| --- | --- | --- | --- | --- | --- |
| rs1059046 | White |  | AA | AC | CC |
|  |  | Case | 19 (0.38) | 22 (0.44) | 9 (0.18) |
|  |  | Control | 15 (0.39) | 17 (0.45) | 6 (0.16) |
|  | Hispanic |  | AA | AC | CC |
|  |  | Case | 1 (0.11) | 5 (0.56) | 3 (0.33) |
|  |  | Control | 0 (0) | 5 (0.62) | 3 (0.38) |
|  | Asian or Pacific Islander |  | AA | AC | CC |
|  |  | Case | 1 (1) | 0 (0) | 0 (0) |
|  |  | Control | 0 (0) | 0 (0) | 1 (1) |
|  | Black, not of Hispanic origin |  | AA | AC | CC |
|  |  | Case | 3 (0.38) | 3 (0.38) | 2 (0.25) |
|  |  | Control | 2 (0.25) | 3 (0.38) | 3 (0.38) |
|  | Other or Unknown (mixed parents) | | AA | AC | CC |
|  |  | Case | 0 (0) | 2 (1) | 0 (0) |
|  |  | Control | 1 (0.5) | 0 (0) | 1 (0.5) |
| rs17886395 | White |  | CC | CG | GG |
|  |  | Case | 0 (0) | 14 (0.28) | 36 (0.72) |
|  |  | Control | 2 (0.05) | 16 (0.42) | 20 (0.53) |
|  | Hispanic |  | CC | CG | GG |
|  |  | Case | 1 (0.11) | 4 (0.44) | 4 (0.44) |
|  |  | Control | 1 (0.12) | 3 (0.38) | 4 (0.5) |
|  | Asian or Pacific Islander |  | CC | CG | GG |
|  |  | Case | 0 (0) | 0 (0) | 1 (1) |
|  |  | Control | 0 (0) | 1 (1) | 0 (0) |
|  | Black, not of Hispanic origin |  | CC | CG | GG |
|  |  | Case | 1 (0.12) | 0 (0) | 7 (0.88) |
|  |  | Control | 0 (0) | 3 (0.38) | 5 (0.62) |
|  | Other or Unknown (mixed parents) | | CC | CG | GG |
|  |  | Case | 0 (0) | 1 (0.5) | 1 (0.5) |
|  |  | Control | 0 (0) | 1 (0.5) | 1 (0.5) |
| rs1965707 | White |  | CC | CT | TT |
|  |  | Case | 28 (0.56) | 19 (0.38) | 3 (0.06) |
|  |  | Control | 21 (0.55) | 15 (0.39) | 2 (0.05) |
|  | Hispanic |  | CC | CT | TT |
|  |  | Case | 4 (0.44) | 5 (0.56) | 0 (0) |
|  |  | Control | 6 (0.75) | 1 (0.12) | 1 (0.12) |
|  | Asian or Pacific Islander |  | CC | CT | TT |
|  |  | Case | 1 (1) | 0 (0) | 0 (0) |
|  |  | Control | 0 (0) | 0 (0) | 1 (1) |
|  | Black, not of Hispanic origin |  | CC | CT | TT |
|  |  | Case | 2 (0.25) | 5 (0.62) | 1 (0.12) |
|  |  | Control | 4 (0.5) | 2 (0.25) | 2 (0.25) |
|  | Other or Unknown (mixed parents) | | CC | CT | TT |
|  |  | Case | 1 (0.5) | 0 (0) | 1 (0.5) |
|  |  | Control | 0 (0) | 1 (0.5) | 1 (0.5) |
| rs1965708 | White |  | AA | AC | CC |
|  |  | Case | 2 (0.04) | 15 (0.3) | 33 (0.66) |
|  |  | Control | 0 (0) | 11 (0.29) | 27 (0.71) |
|  | Hispanic |  | AA | AC | CC |
|  |  | Case | 0 (0) | 2 (0.22) | 7 (0.78) |
|  |  | Control | 1 (0.12) | 1 (0.12) | 6 (0.75) |
|  | Asian or Pacific Islander |  | AA | AC | CC |
|  |  | Case | 0 (0) | 0 (0) | 1 (1) |
|  |  | Control | 0 (0) | 1 (1) | 0 (0) |
|  | Black, not of Hispanic origin |  | AA | AC | CC |
|  |  | Case | 1 (0.12) | 4 (0.5) | 3 (0.38) |
|  |  | Control | 1 (0.12) | 4 (0.5) | 3 (0.38) |
|  | Other or Unknown (mixed parents) | | AA | AC | CC |
|  |  | Case | 0 (0) | 1 (0.5) | 1 (0.5) |
|  |  | Control | 0 (0) | 2 (1) | 0 (0) |
| rs1059047 | White |  | CC | CT | TT |
|  |  | Case | 0 (0) | 9 (0.19) | 38 (0.81) |
|  |  | Control | 0 (0) | 10 (0.28) | 26 (0.72) |
|  | Hispanic |  | CC | CT | TT |
|  |  | Case | 0 (0) | 3 (0.33) | 6 (0.67) |
|  |  | Control | 1 (0.12) | 3 (0.38) | 4 (0.5) |
|  | Asian or Pacific Islander |  | CC | CT | TT |
|  |  | Case | 0 (0) | 0 (0) | 1 (1) |
|  |  | Control | 0 (0) | 0 (0) | 1 (1) |
|  | Black, not of Hispanic origin |  | CC | CT | TT |
|  |  | Case | 0 (0) | 1 (0.12) | 7 (0.88) |
|  |  | Control | 0 (0) | 0 (0) | 8 (1) |
|  | Other or Unknown (mixed parents) | | CC | CT | TT |
|  |  | Case | 0 (0) | 0 (0) | 2 (1) |
|  |  | Control | 0 (0) | 0 (0) | 2 (1) |
| rs1136450 | White |  | CC | CG | GG |
|  |  | Case | 10 (0.21) | 20 (0.43) | 17 (0.36) |
|  |  | Control | 6 (0.17) | 16 (0.44) | 14 (0.39) |
|  | Hispanic |  | CC | CG | GG |
|  |  | Case | 3 (0.33) | 5 (0.56) | 1 (0.11) |
|  |  | Control | 3 (0.38) | 5 (0.62) | 0 (0) |
|  | Asian or Pacific Islander |  | CC | CG | GG |
|  |  | Case | 0 (0) | 0 (0) | 1 (1) |
|  |  | Control | 1 (1) | 0 (0) | 0 (0) |
|  | Black, not of Hispanic origin |  | CC | CG | GG |
|  |  | Case | 2 (0.25) | 4 (0.5) | 2 (0.25) |
|  |  | Control | 1 (0.12) | 4 (0.5) | 3 (0.38) |
|  | Other or Unknown (mixed parents) | | CC | CG | GG |
|  |  | Case | 0 (0) | 2 (1) | 0 (0) |
|  |  | Control | 0 (0) | 2 (1) | 0 (0) |
| rs1136451 | White |  | AA | AG | GG |
|  |  | Case | 32 (0.68) | 14 (0.3) | 1 (0.02) |
|  |  | Control | 22 (0.61) | 12 (0.33) | 2 (0.06) |
|  | Hispanic |  | AA | AG | GG |
|  |  | Case | 3 (0.33) | 5 (0.56) | 1 (0.11) |
|  |  | Control | 2 (0.25) | 3 (0.38) | 3 (0.38) |
|  | Asian or Pacific Islander |  | AA | AG | GG |
|  |  | Case | 1 (1) | 0 (0) | 0 (0) |
|  |  | Control | 0 (0) | 1 (1) | 0 (0) |
|  | Black, not of Hispanic origin |  | AA | AG | GG |
|  |  | Case | 6 (0.75) | 1 (0.12) | 1 (0.12) |
|  |  | Control | 8 (1) | 0 (0) | 0 (0) |
|  | Other or Unknown (mixed parents) | | AA | AG | GG |
|  |  | Case | 1 (0.5) | 1 (0.5) | 0 (0) |
|  |  | Control | 1 (0.5) | 1 (0.5) | 0 (0) |
| rs1059057 | White |  | AA | AG | GG |
|  |  | Case | 39 (0.83) | 8 (0.17) | 0 (0) |
|  |  | Control | 26 (0.72) | 10 (0.28) | 0 (0) |
|  | Hispanic |  | AA | AG | GG |
|  |  | Case | 6 (0.67) | 3 (0.33) | 0 (0) |
|  |  | Control | 4 (0.5) | 3 (0.38) | 1 (0.12) |
|  | Asian or Pacific Islander |  | AA | AG | GG |
|  |  | Case | 1 (1) | 0 (0) | 0 (0) |
|  |  | Control | 1 (1) | 0 (0) | 0 (0) |
|  | Black, not of Hispanic origin |  | AA | AG | GG |
|  |  | Case | 7 (0.88) | 1 (0.12) | 0 (0) |
|  |  | Control | 8 (1) | 0 (0) | 0 (0) |
|  | Other or Unknown (mixed parents) | | AA | AG | GG |
|  |  | Case | 2 (1) | 0 (0) | 0 (0) |
|  |  | Control | 2 (1) | 0 (0) | 0 (0) |
| rs4253527 | White |  | CC | CT | TT |
|  |  | Case | 39 (0.83) | 8 (0.17) | 0 (0) |
|  |  | Control | 30 (0.83) | 6 (0.17) | 0 (0) |
|  | Hispanic |  | CC | CT | TT |
|  |  | Case | 5 (0.56) | 4 (0.44) | 0 (0) |
|  |  | Control | 5 (0.62) | 2 (0.25) | 1 (0.12) |
|  | Asian or Pacific Islander |  | CC | CT | TT |
|  |  | Case | 1 (1) | 0 (0) | 0 (0) |
|  |  | Control | 0 (0) | 1 (1) | 0 (0) |
|  | Black, not of Hispanic origin |  | CC | CT | TT |
|  |  | Case | 6 (0.75) | 2 (0.25) | 0 (0) |
|  |  | Control | 8 (1) | 0 (0) | 0 (0) |
|  | Other or Unknown (mixed parents) | | CC | CT | TT |
|  |  | Case | 1 (0.5) | 1 (0.5) | 0 (0) |
|  |  | Control | 1 (0.5) | 1 (0.5) | 0 (0) |
| rs1130866 | White |  | CC | CT | TT |
|  |  | Case | 13 (0.27) | 22 (0.45) | 14 (0.29) |
|  |  | Control | 8 (0.21) | 22 (0.58) | 8 (0.21) |
|  | Hispanic |  | CC | CT | TT |
|  |  | Case | 1 (0.11) | 5 (0.56) | 3 (0.33) |
|  |  | Control | 3 (0.38) | 5 (0.62) | 0 (0) |
|  | Asian or Pacific Islander |  | CC | CT | TT |
|  |  | Case | 0 (0) | 1 (1) | 0 (0) |
|  |  | Control | 0 (0) | 1 (1) | 0 (0) |
|  | Black, not of Hispanic origin |  | CC | CT | TT |
|  |  | Case | 1 (0.12) | 4 (0.5) | 3 (0.38) |
|  |  | Control | 0 (0) | 2 (0.25) | 6 (0.75) |
|  | Other or Unknown (mixed parents) | | CC | CT | TT |
|  |  | Case | 0 (0) | 2 (1) | 0 (0) |
|  |  | Control | 1 (0.5) | 1 (0.5) | 0 (0) |
| rs4715 | White |  | AA | AC | CC |
|  |  | Case | 6 (0.12) | 19 (0.38) | 25 (0.5) |
|  |  | Control | 2 (0.05) | 12 (0.32) | 23 (0.62) |
|  | Hispanic |  | AA | AC | CC |
|  |  | Case | 1 (0.11) | 3 (0.33) | 5 (0.56) |
|  |  | Control | 0 (0) | 4 (0.5) | 4 (0.5) |
|  | Asian or Pacific Islander |  | AA | AC | CC |
|  |  | Case | 0 (0) | 0 (0) | 1 (1) |
|  |  | Control | 0 (0) | 0 (0) | 1 (1) |
|  | Black, not of Hispanic origin |  | AA | AC | CC |
|  |  | Case | 0 (0) | 2 (0.25) | 6 (0.75) |
|  |  | Control | 0 (0) | 0 (0) | 8 (1) |
|  | Other or Unknown (mixed parents) | | AA | AC | CC |
|  |  | Case | 0 (0) | 0 (0) | 2 (1) |
|  |  | Control | 0 (0) | 1 (0.5) | 1 (0.5) |
| rs1124 | White |  | AA | AG | GG |
|  |  | Case | 10 (0.2) | 24 (0.48) | 16 (0.32) |
|  |  | Control | 2 (0.05) | 16 (0.42) | 20 (0.53) |
|  | Hispanic |  | AA | AG | GG |
|  |  | Case | 1 (0.11) | 4 (0.44) | 4 (0.44) |
|  |  | Control | 0 (0) | 4 (0.5) | 4 (0.5) |
|  | Asian or Pacific Islander |  | AA | AG | GG |
|  |  | Case | 0 (0) | 0 (0) | 1 (1) |
|  |  | Control | 0 (0) | 0 (0) | 1 (1) |
|  | Black, not of Hispanic origin |  | AA | AG | GG |
|  |  | Case | 0 (0) | 2 (0.25) | 6 (0.75) |
|  |  | Control | 0 (0) | 2 (0.25) | 6 (0.75) |
|  | Other or Unknown (mixed parents) | | AA | AG | GG |
|  |  | Case | 0 (0) | 0 (0) | 2 (1) |
|  |  | Control | 1 (0.5) | 0 (0) | 1 (0.5) |
| rs721917 | White |  | CC | CT | TT |
|  |  | Case | 5 (0.1) | 31 (0.62) | 14 (0.28) |
|  |  | Control | 10 (0.26) | 17 (0.45) | 11 (0.29) |
|  | Hispanic |  | CC | CT | TT |
|  |  | Case | 1 (0.11) | 6 (0.67) | 2 (0.22) |
|  |  | Control | 4 (0.5) | 3 (0.38) | 1 (0.12) |
|  | Asian or Pacific Islander |  | CC | CT | TT |
|  |  | Case | 1 (1) | 0 (0) | 0 (0) |
|  |  | Control | 1 (1) | 0 (0) | 0 (0) |
|  | Black, not of Hispanic origin |  | CC | CT | TT |
|  |  | Case | 0 (0) | 6 (0.75) | 2 (0.25) |
|  |  | Control | 2 (0.25) | 1 (0.12) | 5 (0.62) |
|  | Other or Unknown (mixed parents) | | CC | CT | TT |
|  |  | Case | 1 (0.5) | 1 (0.5) | 0 (0) |
|  |  | Control | 0 (0) | 2 (1) | 0 (0) |
| rs2243639 | White |  | AA | AG | GG |
|  |  | Case | 4 (0.08) | 30 (0.6) | 16 (0.32) |
|  |  | Control | 6 (0.16) | 15 (0.41) | 16 (0.43) |
|  | Hispanic |  | AA | AG | GG |
|  |  | Case | 1 (0.11) | 4 (0.44) | 4 (0.44) |
|  |  | Control | 2 (0.25) | 2 (0.25) | 4 (0.5) |
|  | Asian or Pacific Islander |  | AA | AG | GG |
|  |  | Case | 0 (0) | 0 (0) | 1 (1) |
|  |  | Control | 0 (0) | 0 (0) | 1 (1) |
|  | Black, not of Hispanic origin |  | AA | AG | GG |
|  |  | Case | 1 (0.12) | 5 (0.62) | 2 (0.25) |
|  |  | Control | 0 (0) | 2 (0.25) | 6 (0.75) |
|  | Other or Unknown (mixed parents) | | AA | AG | GG |
|  |  | Case | 0 (0) | 0 (0) | 2 (1) |
|  |  | Control | 0 (0) | 0 (0) | 2 (1) |

**Supplementary Table 4 Persistent respiratory morbidity (PRM) *vs* no PRM at 6 months in the univariate and multivariate analysis**

| **Gene** | **SNP** | **Chr** | **Position** | **Allele** | **PRM** | **No PRM** |  | |  | |
| --- | --- | --- | --- | --- | --- | --- | --- | --- | --- | --- |
|  |  |  |  |  | **n (%)** | **n (%)** | **OR (95% CI)** | **p value** | **OR (95% CI)*** | **p value*** |
| *SFTPC* | rs1124 | 8 | AA186 | A | 28 (25) | 52 (37) | 8 (1.5-150) | 0.05 | 11.2 (1.9-217.9) | 0.03 |

Chr = Chromosome, AA = Aminoacid, n (%) = number of the given allele, in parenthesis the percentage of the given allele out of the possible alleles in the particular cohort is shown. *: adjusted for pulmonary dysfunction at discharge and positive bacterial culture.
